# Research on the selection of OTC drug supply chain sales models: based on medical insurance policy

**DOI:** 10.3389/fpubh.2025.1484635

**Published:** 2025-02-19

**Authors:** Heng Wei, Yueyang Liu

**Affiliations:** School of Economics and Management, Shanxi University, Taiyuan, China

**Keywords:** pharmaceutical supply chain, sales model, medical insurance policy, supply chain management, medical pricing

## Abstract

The COVID-19 pandemic has driven a shift toward online medication purchases, prompting major pharmaceutical manufacturers to adopt a dual-channel strategy to enhance competitiveness. This paper examines model selection and pricing challenges for pharmaceutical manufacturers in China’s OTC drug supply chain across different dual-channel models. Our findings indicate that: (1) medical insurance policies significantly enhance profits for pharmaceutical manufacturers and offline retailers; (2) an online direct-selling model yields the highest profit for manufacturers; and (3) increased consumer acceptance of online channels does not necessarily boost demand. Numerical analysis verifies these findings.

## 1 Introduction

With the rapid development of internet technology in the 21st century, internet-based healthcare has encountered unprecedented opportunities and challenges (1). The COVID-19 pandemic has not only exposed the vulnerabilities of the traditional healthcare industry but also increased the demand for more efficient and convenient healthcare services (2). For example, according to data from Zhongkang CMH, online pharmaceutical sales in China grew by 33% during the pandemic, highlighting a significant shift in consumer behavior. In response, the deep integration of internet technology and the healthcare industry has become essential for addressing public health crises (3). Countries and regions, such as the United States, the European Union, and China, have introduced policies to promote the sustainable development of global internet-based healthcare. The United States enacted the Coronavirus Aid, Relief, and Economic Security Act (CARES Act) to strongly promote telemedicine (4). The European Union launched the EU4Health program to advance digital health technologies (5). The Chinese National Health Commission issued the “Opinions on Promoting Internet + Healthcare for the Benefit of the People” to enhance the application of internet technology in healthcare (6). As global medical e-commerce continues to mature, major pharmaceutical manufacturers strive to seize this opportunity. They maintain traditional physical sales channels while actively exploring dual-channel online sales to expand their markets (7). Currently, there are three representative online selling models: online direct selling, online agency selling, and online reselling (8). In the online direct selling model, pharmaceutical manufacturers establish their own online platforms to sell drugs directly to consumers, such as Neptune and Sino Biopharm. The online agency selling model involves manufacturers opening flagship stores on third-party platforms, paying a commission to the platform, which handles the sales, like Tmall. In the online reselling model, manufacturers wholesale drugs to electronic retailers who then own the products and set the prices, such as Alibaba Health and JD.com. Compared to traditional offline sales models, different online sales models have distinct characteristics. Establishing their own platforms requires significant initial investment for pharmaceutical manufacturers but offers the advantage of retaining pricing control, leading to higher profit margins. The online agency selling model can generate significant traffic and increase user engagement through third-party platforms, but the commission paid to the platform reduces overall profits (9). The online reselling model allows manufacturers to wholesale drugs to platforms, avoiding inventory risks and reducing transportation costs (10), but they lose pricing control and market influence, further compressing profit margins. Different healthcare systems approach dual-channel supply chains differently. The U.S. uses a decentralized model, enhancing flexibility and efficiency but facing coordination challenges. China generally employs centralized management, where dual-channel implementation adds complexity, though government policies help stabilize prices and supply. In Europe, supply chain management varies: some countries centralize, while others are more decentralized. Therefore, pharmaceutical manufacturers must comprehensively consider the investment environment, cost, profit potential, and market control of each sales model when choosing an online sales strategy. This consideration is crucial for achieving efficient coordination among all entities in the drug supply chain under different distribution channels and it is a pressing issue in the current digital transformation of the pharmaceutical industry.

The online selling model, with its disintermediation feature, effectively reduces incremental costs across various stages, resulting in lower overall prices for online medications compared to offline prices (11). Unlike traditional dual-channel supply chains, the pharmaceutical market benefits from national medical insurance policies due to its unique characteristics. These policies aim to ensure the accessibility and affordability of medical services and pharmaceutical supply (12), enabling consumers to utilize their personal accounts for medical insurance at designated pharmacies. However, the current medical insurance system primarily applies to offline channels, significantly influencing consumer decision-making. Moreover, according to statistics from a Chinese institution, expenditures from Chinese medical insurance personal accounts constitute approximately 40% of the national pharmaceutical retail market, stabilizing at around 200 billion yuan. This underscores the profound impact of medical insurance policy on the operational management of pharmaceutical retailers. Given these facts, the critical issue facing the dual-channel pharmaceutical supply chain is how to formulate rational medical insurance policies to safeguard the rights and interests of both enterprises and consumers.

In conclusion, this paper is grounded in China’s medical insurance policies and examines the dual-channel supply chain of over-the-counter (OTC) drugs. Three distinct dual-channel pharmaceutical supply chain models have been established, filling a notable gap in the current literature regarding the interaction between medical insurance policies and dual-channel structures specifically for OTC drugs. Through numerical analysis, this study explores the impact of medical insurance policies and consumer channel preferences on drug pricing, demand, and firm performance. This research systematically examines the decision-making processes of pharmaceutical manufacturers within dual-channel OTC drug supply chains shaped by medical insurance policies. We investigate three distinct selling models—online direct selling, online agency selling, and online reselling—each representing different strategic approaches to dual-channel distribution. By modeling the interactions between manufacturers and retailers through the Stackelberg game framework, this study uncovers the unique characteristics and potential challenges of each model. Additionally, it incorporates assumptions grounded in existing research, such as the influence of consumer behavior and medical insurance policies on channel preferences, providing a comprehensive perspective for policymakers aiming to enhance the sustainability and efficiency of pharmaceutical supply chains. It aims to address several key issues:

(1) How should pharmaceutical manufacturers decide among different dual-channel sales models?
(2) How do various medical insurance policies and consumer channel preferences impact pharmaceutical supply chains?
(3) What strategies should the government adopt to formulate scientific, rational, and sustainable medical insurance policies?

## 2 Literature review

### 2.1 Dual-channel supply chain

With the rise of online channel selling models in the supply chain, numerous scholars have conducted research on the dual-channel supply chain, focusing on pricing strategies (13, 14), coordination strategies (15), and the impact of consumer behavior on dual-channel supply chains (16, 17). Regarding pricing strategies for dual-channel supply chains, Liu et al. (18) investigated optimal pricing strategies for manufacturers and retailers with overconfident consumers. Yu et al. (19) compared the impact of consumer strategies on the choice of different sales models and pricing. Huang et al. (20) incorporated production costs into their study and analyzed pricing issues that arise when production costs fluctuate within a dual-channel supply chain. In terms of coordination issues in dual-channel supply chains, Chen et al. (21) constructed a Stackelberg game with the manufacturer as the leader to study coordination schemes in dual channels. Xu et al. (22) examined the impact of coordination contracts on supply chain performance using a mean–variance model, when agents exhibit risk-averse behavior. Mu et al. (23) investigated operational decisions and coordination issues in dual-channel supply chains under credit sales transactions. In studying the impact of consumer behavior on dual-channel supply chains, Sun et al. (24) explored optimal digital showroom strategies for dual-channel supply chains in the presence of online shopping behavior. He et al. (25) focused on closed-loop supply chains and they evaluated the impact of consumer free-riding behavior on product lifecycle carbon emissions in dual-channel closed-loop supply chains. Hu et al. (26) analyzed the influence of consumer delivery time preferences on retailer channel selection and pricing strategies. However, current literature on dual-channel supply chains mostly focuses on ordinary products, with limited research on products with significant social attributes, such as pharmaceuticals.

### 2.2 Pharmaceutical supply chain

Research on pharmaceutical supply chains primarily focuses on operations management (27), pricing strategies (28), and innovative models (29). Within hospital supply chain operations management, Marucheck et al. (30) discuss novel perspectives in tackling drug safety challenges, while Dobrzykowski et al. (31) examine the impact of governmental regulations on pharmaceutical supply chain coordination. Regarding pricing strategies, Chen et al. (32) explore the effects of price ceilings on pharmaceutical pricing decisions, highlighting the negative economic effects of unilateral price ceilings on regulated companies. Building on this research, Chen et al. (33) consider quality regulation factors, finding that appropriate minimum quality standards can enhance the economic performance of the entire drug supply chain. Additionally, Zhang et al. (34) analyze the influence of pharmaceutical enterprise sales channels and various factors on drug quality and pricing using evolutionary game theory, proposing strategies for quality control across different distribution channels. In the realm of pharmaceutical supply chain innovation, Erokhin et al. (35) propose distributed ledger technology to track medical product journeys, improving supply chain models and preventing counterfeit drugs from entering the market. Furthermore, Papalexi et al. (36) evaluate innovation levels in service provision by hospitals and community pharmacies, offering guidance for developing more efficient supply chain strategies for pharmacies and healthcare organizations. While these studies form a solid foundation for understanding pharmaceutical supply chains, they have yet to incorporate medical insurance policies. Gao et al. (37) utilize game theory models to analyze how different healthcare insurance reimbursement strategies, considering reference price effects, impact consumer choices and the medical supply chain online, finding a positive correlation between medical insurance policy and consumer reference price effects. Wen et al. (38), in the context of centralized drug procurement policies, investigate the effects of medical insurance policy on supply chain performance. These studies underscore the importance of integrating medical insurance policies into pharmaceutical supply chain research, particularly within the context of dual-channel supply chains, to reveal the impact of online sales models on pharmaceutical supply chains.

## 3 Problem definition and hypothesis

In this section, we systematically explored the decision-making processes of pharmaceutical manufacturers within the context of dual-channel OTC drug supply chains under medical insurance policies. We introduced and detailed three distinct dual-channel selling models: online direct selling, online agency selling, and online reselling. By employing Stackelberg game theory to model the interactions between manufacturers and retailers, we were able to identify the unique characteristics and challenges associated with each selling model. Furthermore, we established relevant assumptions grounded in existing research, such as consumer behavior and the impact of medical insurance policies on channel preferences. These assumptions form the foundation for our subsequent analysis of pricing mechanisms, demand, and firm performance.

### 3.1 Problem assumptions

**Figure 1:**
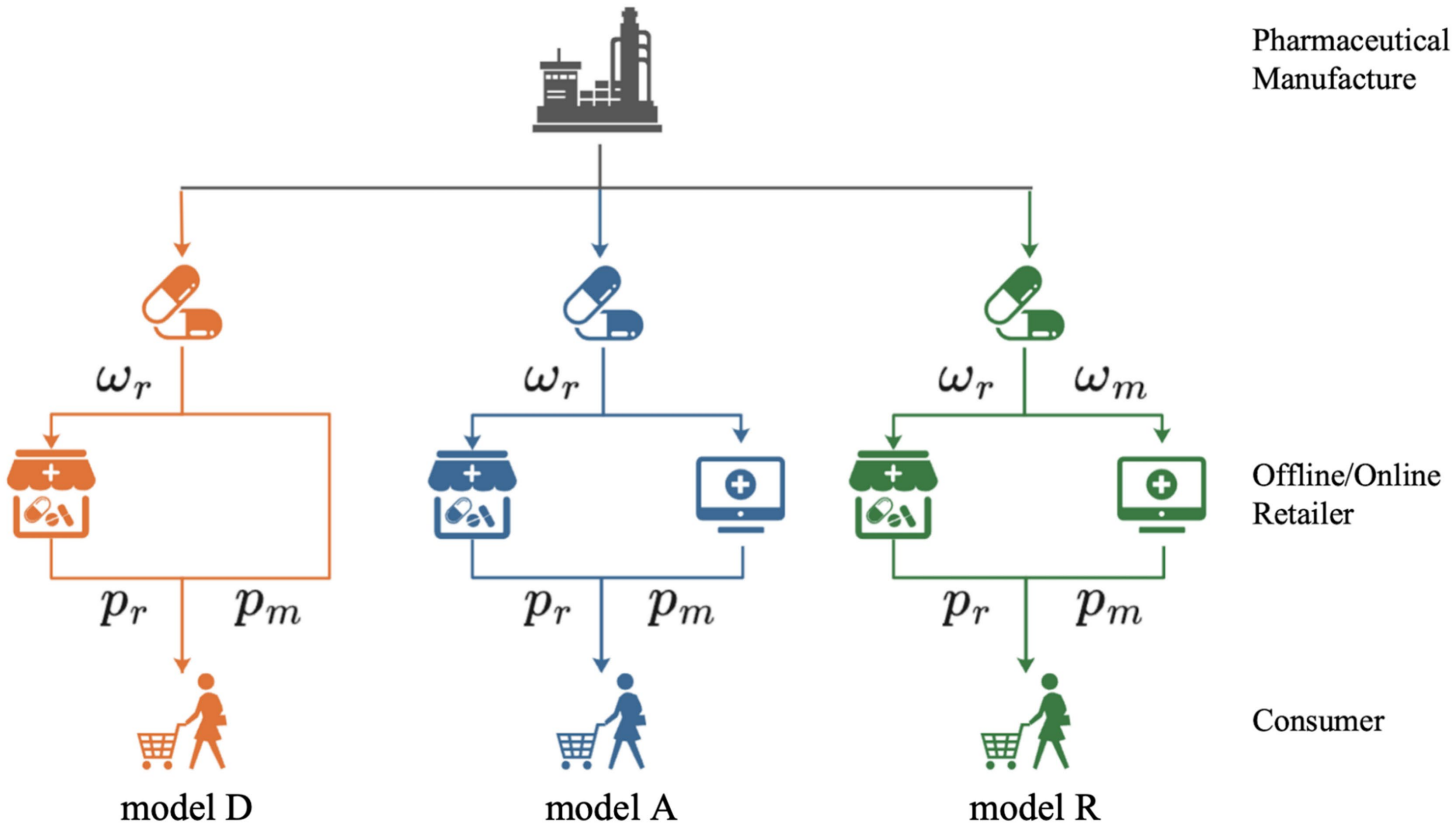
Different dual-channel models.

To study the decision-making processes regarding OTC drug supply chain selling models under medical insurance policy, we examine a dual-channel system led by a pharmaceutical manufacturer. Within this system, the manufacturer develops OTC drugs eligible for medical insurance coverage, distributing them through both traditional and online sales channels. Similar to the study by Liang et al. (39), the interactions between the pharmaceutical manufacturer and the retailers are modeled using the Stackelberg game theory, where the pharmaceutical manufacturer is the Stackelberg leader. This is common in the supply chain literature and in practice. We outline three distinct dual-channel selling models: online direct selling, online agency selling, and online reselling, as shown in Figure 1.

(1) Dual-channel model for online direct selling (model D)

This model consists of a pharmaceutical manufacturer and an offline retailer. In the offline mode, the pharmaceutical manufacturer wholesales OTC drugs to the offline retailer at wholesale prices, allowing the offline retailer to independently set the retail price. In this online mode, the pharmaceutical manufacturer establishes its own platform to sell OTC drugs directly to consumers through the internet.

(2) Dual-channel model for online agency selling (model A)

In this model, alongside the traditional sales approach, pharmaceutical manufacturers collaborate with third-party platforms. These platforms act as intermediaries for drug sales, with the pharmaceutical manufacturers paying a commission $\gamma \;\left(0<\gamma <1\right)$ . In this paper, the commission is treated as an exogenous variable, and it is assumed that the e-commerce platforms do not participate in supply chain decision-making processes.

(3) Dual-channel model for online reselling (model R)

In this model, there is a pharmaceutical manufacturer, an offline retailer, and an online retailer. Both the offline retailer and the online retailer act as distributors for the pharmaceutical manufacturer. The pharmaceutical manufacturer wholesales the products to the offline retailer and the online retailer at different wholesale prices, and both retailers independently determine their retail prices.

### 3.2 Description of relevant assumptions

Based on the supply chain modeling and problem description, the following assumptions are derived from existing research:

*Assumption 1*: Pharmaceutical manufacturers research and develop health-insured over-the-counter (OTC) drugs, such as cold medicine, cough syrup, etc., with pricing determined independently by manufacturers and retailers.

*Assumption 2*: To simplify the model, this paper does not consider costs related to pharmaceutical manufacturers’ self-built platform expenses, drug research, production, logistics, and other aspects.

*Assumption 3*: Consumers are fully rational and will only choose one channel to purchase the drug. Referring to the assumptions of Liu et al. (40), the total market demand for drugs is normalized to 1, and consumers value the drug at $V\;\left(0\leq V\leq 1\right)$ .

*Assumption 4*: Due to restrictions imposed by medical insurance policy, consumers can only use medical insurance in offline channels. Assuming the percentage of consumers’ out-of-pocket expense for drugs is $\lambda \;\left(0<\lambda <1\right)$ , the medical insurance reimbursement ratio is 1−λ .

*Assumption 5*: Similar to the study by Kevin et al. (41), consumer preferences for different channels are converted into their trust levels in these channels. Consumers’ offline acceptance is assumed to be 1, and their online acceptance is $\theta \;\left(0<\theta <1\right)$ .

### 3.3 Description of relevant variables

The relevant variables used in this paper are annotated as shown in Table 1.

**Table 1:** Description of relevant variables.

Notation	Descriptions
$\omega_{r}$	The offline unit wholesale price of pharmaceutical.
$\omega_{m}$	The online unit wholesale price of pharmaceutical.
$p_{r}$	The offline unit retail price of pharmaceutical.
$p_{m}$	The online unit retail price of pharmaceutical.
$D_{r}$	The offline demand of pharmaceutical.
$D_{m}$	The online demand of pharmaceutical.
θ	The consumer acceptance of online purchases, $\theta \in \left(0,1\right)$ .
λ	Percentage of consumers’ out-of-pocket expense, $\lambda \in \left(0,1\right)$ .
γ	Commission per unit charged by online retailers, $\gamma \in \left(0,1\right)$ .
$\pi_{m}$	Pharmaceutical manufacturer’s profit.
$\pi_{r}$	Offline retailer’s profit.
$\pi_{t}$	Online retailer’s profit.

## 4 Model construction and analysis

In this section, we delve into the dual-channel supply chain of over-the-counter (OTC) drugs under the influence of medical insurance policies. The objective is to quantify the impact of different sales models on pricing strategies, consumer behavior, and overall supply chain performance. Building upon the theoretical foundations established in Section 2, we utilize mathematical models to establish three distinct dual-channel models: online direct selling, online agency selling, and online reselling.

### 4.1 The establishment of demand functions

Based on the model proposed by Ryan et al. (42), we assume that consumers value both online and offline pharmaceuticals equally at a value V, where V is a random variable uniformly distributed between 0 and 1. When purchasing through offline channels, consumers buy the drug at a price $p_{r}$ and benefit from a medical insurance reimbursement rate of 1−λ . Thus, the utility for consumers using the offline channel can be expressed as follows:


$$U_{r}=V-\lambda p_{r}$$

For online channels, consumers’ acceptance level is denoted by θ . They purchase the drug online at a price $p_{m}$ . Therefore, the utility for consumers using the online channel can be expressed as follows:


$$U_{m}=\theta V-p_{m}$$

$U_{r}、U_{m}$ measures how much value the consumer gains after accounting for the cost they pay out of pocket. This equation shows the trade-off between the perceived value of the drug and its actual cost to the consumer.

Consumers will demand medication only when the utility from a channel is greater than zero, specifically when $U_{r}>0$ or $U_{m}>0$ . If both utilities are positive, consumers will choose the channel with the higher utility, represented by $\max\left\{U_{r},U_{m}\right\}$ . When $U_{r}=U_{m}$ , indicating equal utility from both channels, consumers’ valuation of the medication $V^{*}$ is calculated as $V^{*}=\frac{\lambda p_{r}-p_{m}}{1-\theta }$ .

(1) When the online retail price $p_{r}$ of the medication satisfies $p_{r}<\frac{p_{m}}{\theta \lambda }$ , the online price is perceived by consumers as relatively high compared to their expectations. Consequently, consumers will not choose the online channel to purchase the medication. According to the principle of utility, consumers will not purchase the medication when their valuation V falls within the range $V\in \left[0,V_{r}\right]$ ; they will opt for the offline channel when V is within $V\in \left[V_{r},1\right]$ . In this scenario, the demand for the medication through offline channels and online channels, respectively, is $D_{r}=1-\lambda p_{r}$ and $D_{m}=0$ .
(2) When the online retail price $p_{r}$ of the drug satisfies $p_{r}>\frac{p_{m}}{\theta \lambda }$ , there is demand for the drug both online and offline. According to utility theory, when consumers’ utility valuation of the drug V falls within intervals $V\in \left[0,V_{m}\right]$ , they do not purchase the drug. Within the intervals $V\in \left[V_{m},V^{*}\right]$ , consumers opt for the online channel, and within intervals $V\in \left[V^{*},1\right]$ , they choose the offline channel. The demand for the drug is $D_{r}=\frac{1-\theta -\lambda p_{r}+p_{m}}{1-\theta }$ offline and $D_{m}=\frac{\lambda \theta p_{r}-p_{m}}{\theta \left(1-\theta \right)}$ online.

This paper focuses exclusively on the second scenario, where the drug competes in both offline and online channels within the market.

### 4.2 Dual-channel model for online direct selling

In the dual-channel model for online direct selling, the decision sequence among supply chain members is as follows: the pharmaceutical manufacturer first determines the offline wholesale price $\omega_{r}$ and the online retail price $p_{m}$ . The offline retailer, as a follower in the supply chain, subsequently decides the offline retail price $p_{r}$ . The profit functions for the pharmaceutical manufacturer and the offline retailer are in Equations 1, 2:


(1) $$\pi_{m}^{D}\left(\omega_{r}^{D},p_{m}^{D}\right)=\omega_{r}^{D}D_{r}+p_{m}^{D}D_{m}$$

(2) $$\pi_{r}^{D}\left(p_{r}^{D}\right)=\left(p_{r}^{D}-\omega_{r}^{D}\right)D_{r}$$

The profit formula for pharmaceutical manufacturers consists of two parts: the first part represents the profit from online sales through the manufacturers’ self-built platforms, and the second part represents the profit from supplying drugs to offline retailers.

Using backward induction to solve the model, we begin by addressing the two-stage decision-making problem. Backward induction is a method commonly used in dynamic optimization to solve problems by first analyzing the final stage of the decision process and then working backward to determine the optimal strategies for earlier stages. Specifically, we first analyze the second stage, where consumers make their purchasing decisions based on given prices and other parameters. Once the optimal outcomes for the second stage are established, we move to the first stage, where firms or decision-makers set strategies such as pricing or reimbursement rates, taking into account the expected behavior of consumers in the subsequent stage. This approach ensures that the solutions are both consistent and optimal across the entire decision-making framework.

#### 4.2.1 Proposition 1

When $8\lambda -\theta \left(1+6\lambda +\lambda^{2}\right)>0$ , the profit function $\pi_{m}^{D}$ with respect to $\left(\omega_{r}^{D},p_{m}^{D}\right)$ is strictly jointly differentiable. In this case, the optimal Nash equilibrium for both the pharmaceutical manufacturer and the offline retailer is $\left({\omega_{r}^{D}}^{*},{p_{r}^{D}}^{*},{p_{m}^{D}}^{*}\right)$ , with optimal profits $\left({\pi_{m}^{D}}^{*},{\pi_{r}^{D}}^{*}\right)$ . The demands in the online and offline channels are $\left({D_{r}^{D}}^{*},{D_{m}^{D}}^{*}\right)$ .

Proof see Appendix A.


$$\left\{ \begin{array}{c} {\omega_{r}^{D}}^{*}=\frac{\left(-1+\theta \right)\left(4-\theta +\theta \lambda \right)}{-8\lambda +\theta \left(1+6\lambda +\lambda^{2}\right)}\\ {p_{r}^{D}}^{*}=\frac{2\left(3-4\theta +\theta^{2}\right)}{-8\lambda +\theta \left(1+6\lambda +\lambda^{2}\right)}\\ {p_{m}^{D}}^{*}=\frac{\left(-1+\theta \right)\left(\theta +3\theta \lambda \right)}{-8\lambda +\theta \left(1+6\lambda +\lambda^{2}\right)} \end{array}\right.$$

$$\left\{ \begin{array}{c} {D_{r}^{D}}^{*}=\frac{\lambda \left(-2+\theta +\theta \lambda \right)}{-8\lambda +\theta \left(1+6\lambda +\lambda^{2}\right)}\\ {D_{m}^{D}}^{*}=\frac{1-3\lambda +2\theta \lambda }{-8\lambda +\theta \left(1+6\lambda +\lambda^{2}\right)} \end{array}\right.$$

$$\left\{ \begin{array}{c} {\pi_{m}^{D}}^{*}=\frac{\left(-1+\theta \right)\left(1+\theta \lambda \right)}{-8\lambda +\theta \left(1+6\lambda +\lambda^{2}\right)}\\ {\pi_{r}^{D}}^{*}=-\frac{\left(-1+\theta \right)\lambda {\left(-2+\theta +\theta \lambda \right)}^{2}}{{\left(-8\lambda +\theta \left(1+6\lambda +\lambda^{2}\right)\right)}^{2}} \end{array}\right.$$

### 4.3 Dual-channel model for online agency selling

In the online agency selling model, the decision sequence is as follows: pharmaceutical manufacturers first determine the offline wholesale price $\omega_{r}$ and the online retail price $p_{m}$ . Based on these decisions, offline retailers determine the offline retail price $p_{r}$ . The online retailer does not participate in the decision-making process. The profit functions for the pharmaceutical manufacturer, offline retailer, and online retailer are given in Equations 3–5:


(3) $$\pi_{m}^{A}\left(\omega_{r}^{A},p_{m}^{A}\right)=\omega_{r}^{A}D_{r}+\left(1-\gamma \right)p_{m}^{A}D_{m}$$

(4) $$\pi_{r}^{A}\left(p_{r}^{A}\right)=\left(p_{r}^{A}-\omega_{r}^{A}\right)D_{r}$$

(5) $$\pi_{t}^{A}=\gamma p_{m}^{A}D_{m}$$

The profit formula for pharmaceutical manufacturers is divided into two parts: the first part represents the profit from online sales through third-party platforms, and the second part represents the profit from supplying drugs to offline retailers.

#### 4.3.1 Proposition 2

When $-4\left(-1+\gamma \right)\left(-2+\theta \right)\lambda +\theta {\left(1+\lambda -\gamma \lambda \right)}^{2}<0$ , the profit function $\pi_{m}^{A}$ with respect to $\left(\omega_{r}^{A},p_{m}^{A}\right)$ is strictly jointly differentiable. At this optimal equilibrium point $\left({\omega_{r}^{A}}^{*},{p_{r}^{A}}^{*},{p_{m}^{A}}^{*}\right)$ , the optimal profits for the pharmaceutical manufacturer, offline retailer, and online retailer are $\left({\pi_{m}^{A}}^{*},{\pi_{r}^{A}}^{*},{\pi_{t}^{A}}^{*}\right)$ respectively. The offline and online demands are $\left({D_{r}^{A}}^{*},{D_{m}^{A}}^{*}\right)$ .

Proof see Appendix B.


$$\left\{ \begin{array}{c} {\omega_{r}^{A}}^{*}=\frac{\left(-1+\gamma \right)\left(-1+\theta \right)\left(-4+\theta +\left(-1+\gamma \right)\theta \lambda \right)}{\left(-1+\gamma \right)\lambda \left(\theta \lambda \left(-1+\gamma \right)-6\theta +8\right)+\theta }\\ {p_{r}^{A}}^{*}=\frac{2\left(-1+\gamma \right)\left(-3+\theta \right)\left(-1+\theta \right)}{\left(-1+\gamma \right)\lambda \left(\theta \lambda \left(-1+\gamma \right)-6\theta +8\right)+\theta }\\ {p_{m}^{A}}^{*}=-\frac{\left(-1+\theta \right)\left(-\theta -3\theta \lambda +3\gamma \theta \lambda \right)}{\left(-1+\gamma \right)\lambda \left(\theta \lambda \left(-1+\gamma \right)-6\theta +8\right)+\theta } \end{array}\right.$$

$$\left\{ \begin{array}{c} {D_{r}^{A}}^{*}=\frac{\left(-1+\gamma \right)\lambda \left(2+\theta \left(-1+\left(-1+\gamma \right)\lambda \right)\right)}{\left(-1+\gamma \right)\lambda \left(\theta \lambda \left(-1+\gamma \right)-6\theta +8\right)+\theta }\\ {D_{m}^{A}}^{*}=\frac{1-\left(-1+\gamma \right)\left(-3+2\theta \right)\lambda }{\left(-1+\gamma \right)\lambda \left(\theta \lambda \left(-1+\gamma \right)-6\theta +8\right)+\theta } \end{array}\right.$$

$$\left\{ \begin{array}{c} {\pi_{m}^{A}}^{*}=\frac{\left(-1+\gamma \right)\left(-1+\theta \right)\left(-1+\left(-1+\gamma \right)\theta \lambda \right)}{\left(-1+\gamma \right)\lambda \left(\theta \lambda \left(-1+\gamma \right)-6\theta +8\right)+\theta }\\ {\pi_{r}^{A}}^{*}=-\frac{{\left(-1+\gamma \right)}^{2}\left(-1+\theta \right)\lambda {\left(2+\theta \left(-1+\left(-1+\gamma \right)\lambda \right)\right)}^{2}}{{\left(\left(-1+\gamma \right)\lambda \left(\theta \lambda \left(-1+\gamma \right)-6\theta +8\right)+\theta \right)}^{2}}\\ {\pi_{t}^{A}}^{*}=\frac{\gamma \left(-1+\theta \right)\theta \left(-1+3\left(-1+\gamma \right)\lambda \right)\left(-1+\left(-1+\gamma \right)\left(-3+2\theta \right)\lambda \right)}{{\left(\left(-1+\gamma \right)\lambda \left(\theta \lambda \left(-1+\gamma \right)-6\theta +8\right)+\theta \right)}^{2}} \end{array}\right.$$

### 4.4 Dual-channel model for online reselling

In this model, the decision sequence among supply chain members is as follows: pharmaceutical manufacturers first determine the offline wholesale price $\omega_{r}$ and the online wholesale price $\omega_{m}$ . Subsequently, offline retailer and online retailer independently decide on the offline retail price $p_{r}$ and the online retail price $p_{m}$ . The profit functions for the pharmaceutical manufacturer, offline retailer, and online retailer are are given in Equations 6–8:


(6) $$\pi_{m}^{R}\left(\omega_{r}^{R},\omega_{m}^{R}\right)=\omega_{r}^{R}D_{r}+\omega_{m}^{R}D_{m}$$

(7) $$\pi_{r}^{R}\left(p_{r}^{R}\right)=\left(p_{r}^{R}-\omega_{r}^{R}\right)D_{r}$$

(8) $$\pi_{t}^{R}\left(p_{m}^{R}\right)=\left(p_{m}^{R}-\omega_{m}^{R}\right)D_{m}$$

The profit formula for pharmaceutical manufacturers consists of two parts: the first part represents the profit from supplying drugs to online retailers, and the second part represents the profit from supplying drugs to offline retailers.

#### 4.4.1 Proposition 3

When $16\lambda +4\theta^{2}\lambda -\theta \left(1+18\lambda +\lambda^{2}\right)>0$ , the profit function $\pi_{m}^{R}$ with respect to $\left(\omega_{r}^{R},\omega_{m}^{R}\right)$ is strictly jointly differentiable. At this optimal equilibrium point $\left({\omega_{r}^{R}}^{*},{p_{r}^{R}}^{*},{p_{m}^{R}}^{*}\right)$ , the optimal profits for the pharmaceutical manufacturer, offline retailer, and online retailer are $\left({\pi_{m}^{R}}^{*},{\pi_{r}^{R}}^{*},{\pi_{t}^{R}}^{*}\right)$ respectively. The offline and online demands are $\left({D_{r}^{R}}^{*},{D_{m}^{R}}^{*}\right)$ .

Proof see Appendix C.


$$\left\{ \begin{array}{c} {\omega_{r}^{R}}^{*}=-\frac{\left(-1+\theta \right)\left(8+\theta \left(-3+\lambda \right)\right)}{16\lambda +4\theta^{2}\lambda -\theta \left(1+18\lambda +\lambda^{2}\right)}\\ {\omega_{m}^{R}}^{*}=\frac{2\left(-1+\theta \right)\theta \left(-1+\left(-3+\theta \right)\lambda \right)}{16\lambda +4\theta^{2}\lambda -\theta \left(1+18\lambda +\lambda^{2}\right)}\\ {p_{r}^{R}}^{*}=\frac{6\left(2-3\theta +\theta^{2}\right)}{16\lambda +4\theta^{2}\lambda -\theta \left(1+18\lambda +\lambda^{2}\right)}\\ {p_{m}^{R}}^{*}=\frac{\left(-1+\theta \right)\theta \left(-1+\left(-9+4\theta \right)\lambda \right)}{16\lambda +4\theta^{2}\lambda -\theta \left(1+18\lambda +\lambda^{2}\right)} \end{array}\right.$$

$$\left\{ \begin{array}{c} {D_{r}^{R}}^{*}=\frac{\lambda \left(-4+\theta \left(3+\lambda \right)\right)}{-16\lambda -4\theta^{2}\lambda +\theta \left(1+18\lambda +\lambda^{2}\right)}\\ {D_{m}^{R}}^{*}=\frac{1-3\lambda +2\theta \lambda }{\theta -16\lambda +18\theta \lambda -4\theta^{2}\lambda +\theta \lambda^{2}} \end{array}\right.$$

$$\left\{ \begin{array}{c} {\pi_{m}^{R}}^{*}=-\frac{\left(-1+\theta \right)\left(2+\theta \lambda \right)}{16\lambda +4\theta^{2}\lambda -\theta \left(1+18\lambda +\lambda^{2}\right)}\\ {\pi_{r}^{R}}^{*}=-\frac{\left(-1+\theta \right)\lambda {\left(-4+\theta \left(3+\lambda \right)\right)}^{2}}{{\left(16\lambda +4\theta^{2}\lambda -\theta \left(1+18\lambda +\lambda^{2}\right)\right)}^{2}}\\ {\pi_{t}^{R}}^{*}=-\frac{\left(-1+\theta \right)\theta {\left(1+\left(-3+2\theta \right)\lambda \right)}^{2}}{{\left(16\lambda +4\theta^{2}\lambda -\theta \left(1+18\lambda +\lambda^{2}\right)\right)}^{2}} \end{array}\right.$$

## 5 Analysis of the results

In this section, we analyze and discuss the numerical results obtained from our study on the dual-channel supply chain of over-the-counter (OTC) drugs under the influence of medical insurance policies. Building upon the methodologies and theoretical framework outlined in Section 2 and 3, we present findings that elucidate the effects of different sales models—online direct selling, online agency selling, and online reselling—on pricing strategies, channel demand, and overall supply chain performance.

### 5.1 The relationship between drug prices, demand, and profit

#### 5.1.1 Theorem 1

By calculating the above model, we derive the relationships between drug prices, channel demand, and corporate profits as follows:


$$p_{r}-p_{m}>0.\;D_{r}-D_{m}>0.\pi_{m}-\pi_{r}>0.$$

Proof see Appendix D.

Theorem 1 elucidates the relationships among retail prices, channel demand, and supply chain member profits across different selling models. Specifically, offline retail prices for drugs are generally higher than online retail prices, and offline channel demand typically surpasses online demand. Pharmaceutical manufacturers generally achieve higher profits compared to offline retailers. This phenomenon arises because online channels reduce the traditional distribution layers and markups associated with offline channels, resulting in lower overall drug prices online. However, due to the unique nature of pharmaceuticals and constraints imposed by medical insurance policies, most consumers still prefer purchasing drugs through traditional offline channels despite the more favorable online pricing. Consequently, demand in the offline channel generally exceeds that in the online channel. Pharmaceutical manufacturers, as leaders in the supply chain, thus enjoy higher profits compared to offline retailers.

### 5.2 The impact of medical insurance policy on sales model

#### 5.2.1 Theorem 2

Theorem 2 describes the impact of consumers’ out-of-pocket percentage in medical insurance on pricing decisions, channel demands, and supply chain member profits.

(1)


$$\frac{\partial \omega_{r}}{\partial \lambda }<0.\frac{\partial p_{r}}{\partial \lambda }<0.\frac{\partial p_{m}}{\partial \lambda }<0$$

(2)


$$\frac{\partial D_{r}}{\partial \lambda }\langle 0.\frac{\partial D_{m}}{\partial \lambda }\rangle 0$$

(3)


$$\frac{\partial \pi_{m}}{\partial \lambda }<0.\frac{\partial \pi_{r}}{\partial \lambda }<0$$

Proof see Appendix E.

Theorem 2 (1) indicates that the offline wholesale price, offline retail price, and online retail price of drugs decrease as the percentage of consumers’ out-of-pocket increases. This trend arises because medical insurance usage is currently restricted to offline channels. As the percentage of consumers’ out-of-pocket increases, consumer face greater economic burdens when purchasing drugs, leading more consumers to prefer online channels and intensifying competition between channels. Consequently, offline retailers, in order to compete for market share, must reduce offline retail prices. Simultaneously, the decreased purchasing power of consumers pressures pharmaceutical manufacturers to lower both wholesale prices for drugs and online retail prices to maintain their profits. Theorem 2 (2) illustrates that offline channel demand decreases with an increase in the percentage of consumers’ out-of-pocket, while online demand increases. This shift occurs because as the percentage of consumers’ out-of-pocket rises, price-sensitive consumers opt to purchase drugs through online channels, thereby reducing demand in offline channels. Theorem 2 (3) notes that both pharmaceutical manufacturers and offline retailer profits decrease with an increase in the percentage of consumers’ out-of-pocket. This outcome arises from continuous reductions in drug prices, ultimately diminishing overall profitability for both entities. In conclusion, raising the medical insurance reimbursement ratio benefits consumers, pharmaceutical manufacturers, and offline retailers alike. However, it can adversely affect consumers who do not benefit from medical insurance coverage and negatively impact drug pricing dynamics. Therefore, governments should establish an appropriate reimbursement ratio to balance the interests of all parties involved.

### 5.3 The impact of consumer online acceptance on sales model

#### 5.3.1 Theorem 3

Theorem 3 elucidates the influence of consumer online acceptance on pricing decisions, channel demands, and supply chain member profits.

(1) When $\theta <A^{i}$ , $\frac{\partial \omega_{r}^{i}}{\partial \theta }<0$ , otherwise $\frac{\partial \omega_{r}^{i}}{\partial \theta }>0$ . $\frac{\partial p_{r}}{\partial \theta }\langle 0.\frac{\partial p_{m}}{\partial \theta }\rangle 0$

(2)


$$\frac{\partial D_{r}}{\partial \theta }>0.\frac{\partial D_{m}}{\partial \theta }<0$$

(3)


$$\frac{\partial \pi_{m}}{\partial \theta }>0.\frac{\partial \pi_{r}}{\partial \theta }<0$$

$$A^{D}=\frac{2\left(4\lambda +\sqrt{1+3\lambda -\lambda^{2}-3\lambda^{3}}\right)}{1+6\lambda +\lambda^{2}}$$

$$\begin{array}{l} A^{A}=\\ \frac{16\lambda -16\gamma \lambda +\sqrt{{\left(-16\lambda +16\gamma \lambda \right)}^{2}-4\left(-4+12\lambda -12\gamma \lambda \right)\left(1+6\lambda -6\gamma \lambda +\lambda^{2}-2\gamma \lambda^{2}+\gamma^{2}\lambda^{2}\right)}}{2\left(1+6\lambda -6\gamma \lambda +\lambda^{2}-2\gamma \lambda^{2}+\gamma^{2}\lambda^{2}\right)} \end{array}$$

$$A^{R}=\frac{2\left(8\lambda +\sqrt{6}\sqrt{1+\lambda -\lambda^{2}-\lambda^{3}}\right)}{3+12\lambda +\lambda^{2}},i=D,A,R$$

Proof see Appendix F.

Theorem 3 (1) indicates that as consumer online acceptance increases from a low level, offline wholesale drug prices initially decrease, but beyond a certain threshold, offline wholesale prices begin to rise. Conversely, offline retail prices exhibit a negative correlation with online acceptance, while online retail prices show a positive correlation. This occurs because pharmaceutical manufacturers adjust online retail prices to maximize profits as consumer preference for online channel grows, while offline retailer reduces offline retails price to compete. Consequently, this decision-making affects procurement pressures, prompting pharmaceutical manufacturers to lower wholesale price. However, excessively low wholesale prices impact manufacturer profits, causing them to increase wholesale prices. Notably, Theorem 3 (2) reveals a positive correlation between offline channel demand and consumer online acceptance, whereas online channel demand correlates negatively with consumer online acceptance. With increasing consumer preference for online channels, online retail prices rise, leading to reduced overall online channel demand. Consequently, offline retail prices decrease, prompting more consumers to choose offline drug purchases, thereby increasing offline demand. Theorem 3 (3) indicates that pharmaceutical manufacturers’ profits increase with rising consumer online acceptance, whereas offline retailers’ profits decline. Despite reduced online demand due to higher online retail drug prices, pharmaceutical manufacturers achieve overall profit gains. In contrast, offline retailers experience lower profits as more consumers opt for online drug purchases, despite lower offline retail prices.

## 6 Numerical study

Section 5 conducts a numerical study to empirically examine the dual-channel supply chain dynamics for over-the-counter (OTC) drugs under varying medical insurance policies. Through numerical simulations and mathematical modeling, this section quantifies the effects of different sales models—online direct selling, online agency selling, and online reselling—on pricing strategies, channel demand, and overall supply chain performance.

### 6.1 The impact of medical insurance policies on pricing, channel demand, and profits

**Figure 2:**
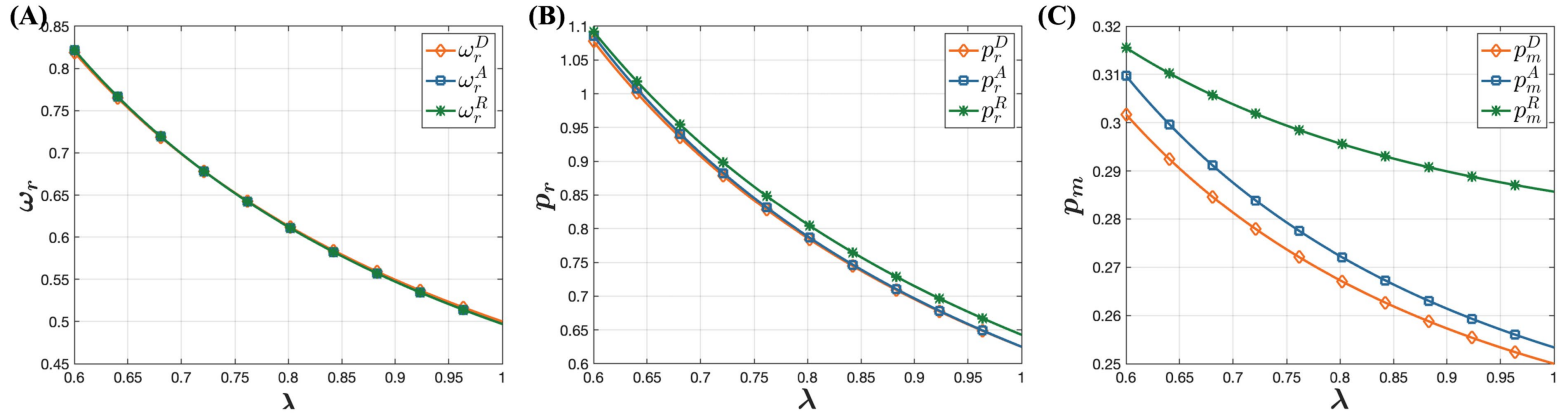
The influence of medical insurance policies on pricing under the D, A, and R models. **(A)** The influence of medical insurance policies on offline wholesale price. **(B)** The influence of medical insurance policies on offline retail price. **(C)** The influence of medical insurance policies on online retail price.

**Figure 3:**
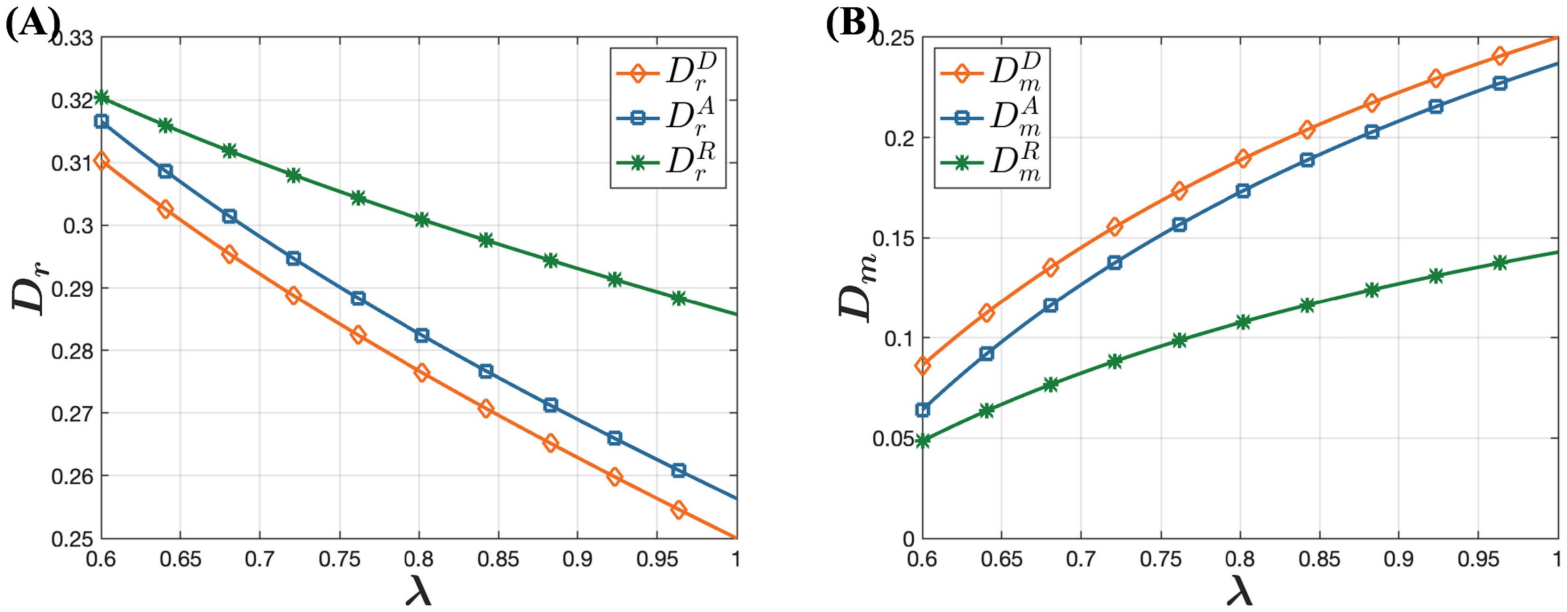
The influence of medical insurance policies on demand under the D, A, and R models. **(A)** The influence of medical insurance policies on offline demand. **(B)** The influence of medical insurance policies on online demand.

**Figure 4:**
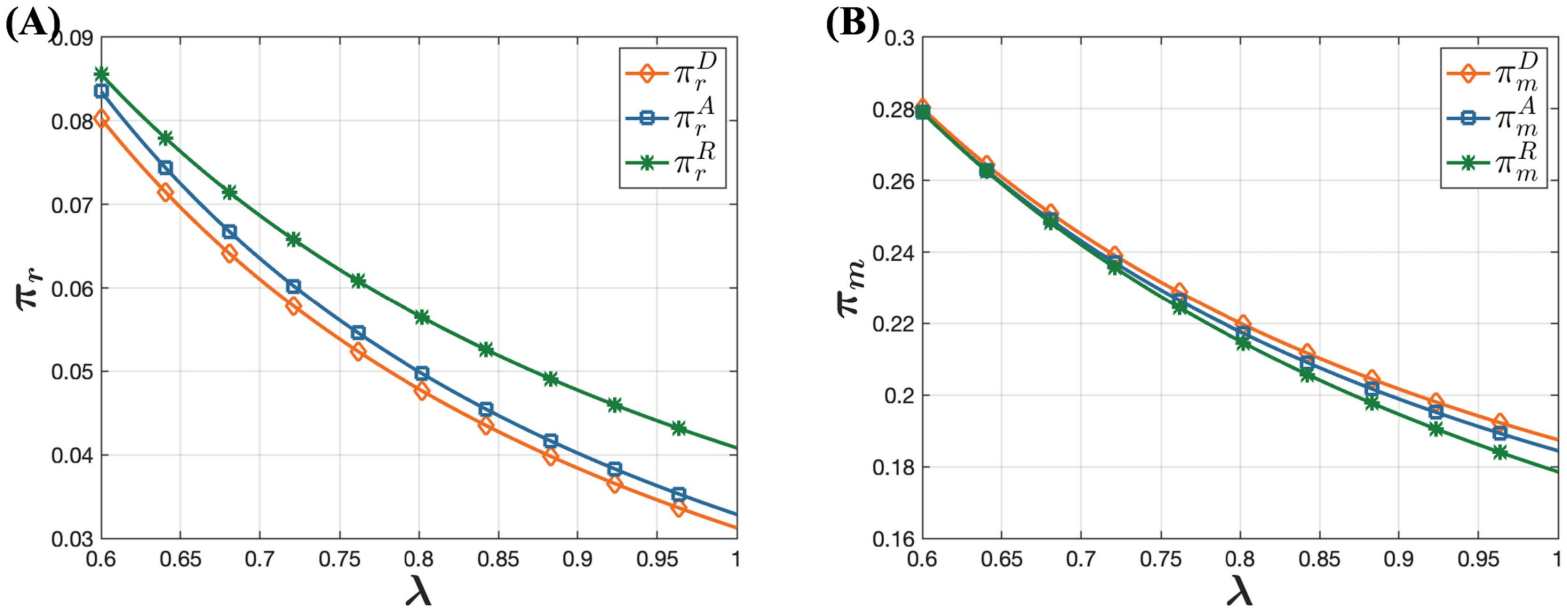
The influence of medical insurance policies on profitability under the D, A, and R models. **(A)** The influence of medical insurance policies on pharmaceutical manufacturer’s profit. **(B)** The influence of medical insurance policies on offline retailer’s profit.

In this section, numerical analysis is employed to validate the aforementioned properties and explore the impact of varying percentages of consumers’ out-of-pocket expenses on drug pricing decisions, channel demands, and supply chain member profits under the D, A, and R models. The parameters used are set as θ=0.5 and γ=0.05 . These parameter settings are verified to satisfy the constraint conditions of the model’s optimal solutions. Additionally, λ is assumed to range between 0.6 and 1, within which the optimal solution conditions are also met. The software used for simulation is MATLAB 2018a. Detailed simulation results are presented in Figures 2–4.

From Figure 2, it is evident that under the D, A, R sales models, offline wholesale prices, offline retail prices, and online retail prices of drugs all decrease with increasing percentage of consumers’ out-of-pocket expenses. This trend aligns with the earlier discussions suggesting that higher percentage of consumers’ out-of-pocket expenses may increase the financial burden on consumer for purchasing drugs offline, thereby reducing demand for offline drug purchases. The decline in channel demand directly affects the purchasing needs of drug retailers, forming a closely interconnected chain. Specifically, as the percentage of consumers’ out-of-pocket expenses increases, offline retailers reduce drug retail prices to maintain their profits and attract consumers. Despite this reduction, consumer demand for the offline channel still decreases. In response, pharmaceutical manufacturers lower offline wholesale prices to maintain market share. This adjustment cascades through the market, prompting offline retailers to further reduce prices and necessitating promotional pricing strategies for online wholesale drug prices. This phenomenon reflects the competitive market environment, where offline retailers lowering prices triggers adjustments in prices across other channels. These findings underscore the profound impact of medical insurance policies on drug market prices, offering insights into the mechanisms shaping drug pricing.

Figure 3 illustrates the impact of varying percentages of consumers’ out-of-pocket expenses on the demand for different types of channels. Specifically, it shows that offline channel demand decreases as the percentage of consumers’ out-of-pocket expenses increases, while online channel demand increases with an increase in the percentage of consumers’ out-of-pocket expenses. This observation is consistent with the earlier conclusions. As the percentage of consumers’ out-of-pocket expenses rises, consumers face greater financial burdens when purchasing drugs offline. Consequently, more consumers are shifting toward online channels to purchase drugs, resulting in decreased offline channel demand and increased online channel demand. This shift underscores the significant influence of medical insurance policies on consumer purchasing behavior and channel preferences within the pharmaceutical market.

Based on Figure 4, it is clear that as the percentage of consumers’ out-of-pocket expenses increases, both pharmaceutical manufacturers and offline retailers experience declining profits. Pharmaceutical manufacturers are directly affected by the decreasing prices in both offline and online retail markets. Even though there is a rise in online channel demand, it does not fully offset the income loss from reduced online retail prices, creating a significant profitability challenge. Offline retailers also face profitability issues as the medical insurance out-of-pocket ratio rises. Higher consumer costs result in increased price sensitivity, leading to decreased offline channel demand. To maintain market share, pharmaceutical manufacturers may further reduce retail prices, adversely affecting offline retailers’ profits. Consequently, offline retailers are compelled to explore alternative sales models to sustain their profitability. In conclusion, reducing the percentage of consumers’ out-of-pocket expenses and increasing drug reimbursement rates positively impact both pharmaceutical manufacturers and offline retailers. Therefore, governments should develop suitable medical insurance policies to ensure the sustainability of the healthcare system and enhance citizens’ medical security.

### 6.2 The impact of consumer online purchases acceptance on pricing, channel demand, and profits

**Figure 5:**
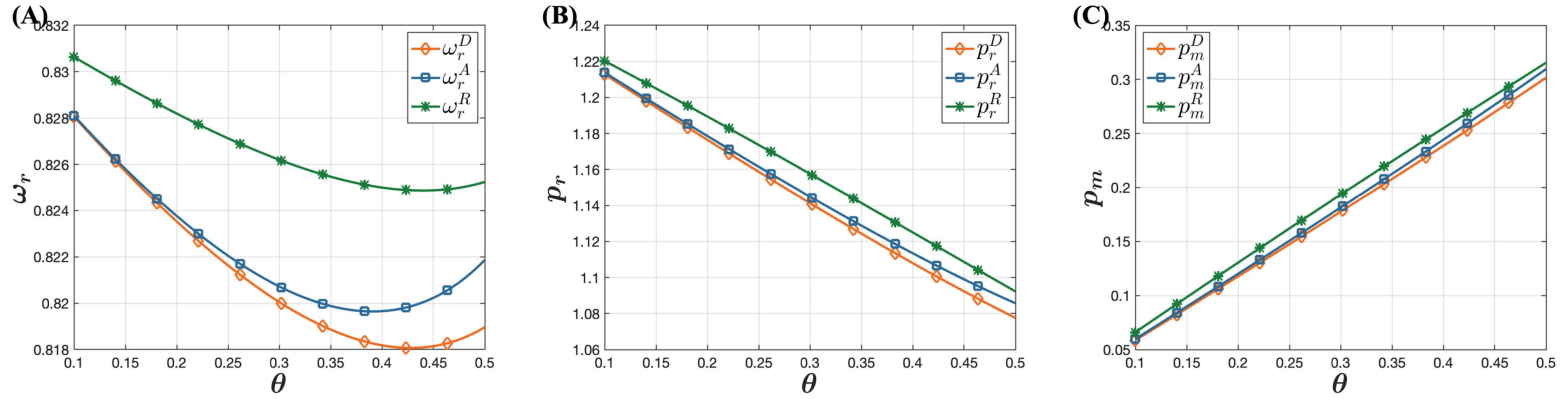
The Influence of Consumer Acceptance of Online Purchases on Dual-Channel Pricing Strategies. **(A)** The influence of consumer acceptance of online purchases on offline wholesale price. **(B)** The influence of consumer acceptance of online purchases on offline retail price. **(C)** The influence of consumer acceptance of online purchases on online retail price.

**Figure 6:**
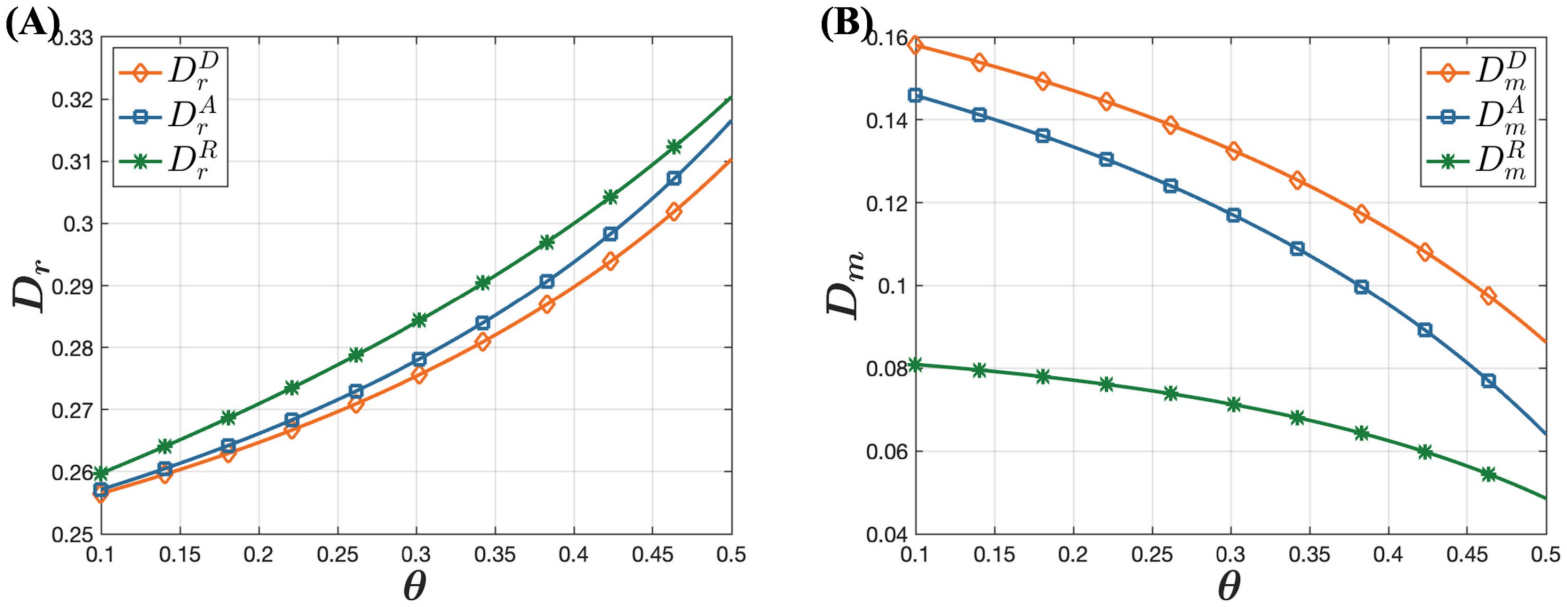
The influence of consumer acceptance of online purchases on demand under the D, A, and R models. **(A)** The influence of consumer acceptance of online purchases on offline demand. **(B)** The influence of consumer acceptance of online purchases on online demand.

**Figure 7:**
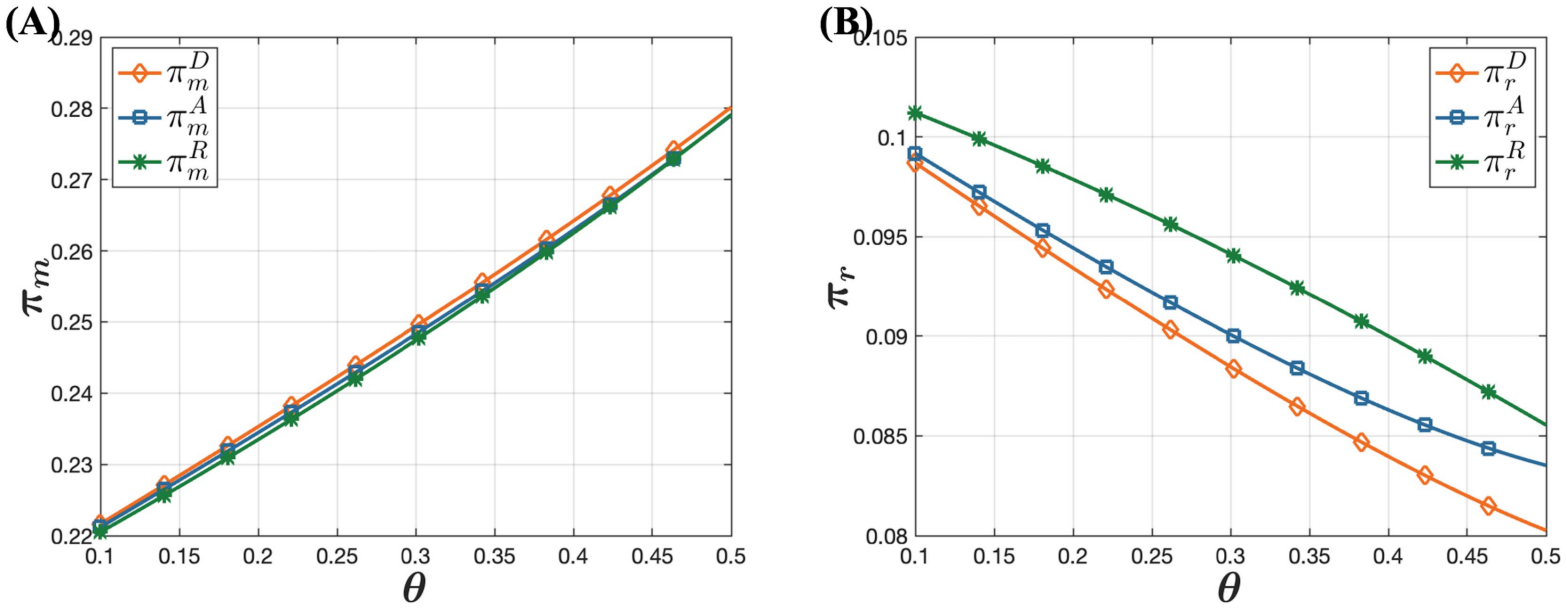
The influence of consumer acceptance of online purchases on profitability under the D, A, and R models. **(A)** The influence of consumer acceptance of online purchases on pharmaceutical manufacturer’s profit. **(B)** The influence of consumer acceptance of online purchases on offline retailer’s profit.

In this section, we investigate the impact of online channel trustworthiness on drug pricing decisions, channel demands, and supply chain member profits under the D, A, and R models. The parameters are set as λ=0.6 , γ=0.05 , and θ ranges from 0.1 to 0.5. The simulation results are presented in Figures 5–7.

In Figure 5, it shows that consumer acceptance of online purchases has minimal impact on drug wholesale prices, which initially decrease and then increase. However, offline retail prices of drugs decline with rising trust in online channels, while online retail prices increase. This occurs because as more consumers prefer purchasing drugs through online channels, offline retailers face heightened market competition. To maintain their market share and profitability, offline retailers implement strategies like promotions and discounts, reducing offline retail prices. This approach attracts price-sensitive consumers back to physical stores, enhancing the competitiveness and viability of offline retailers in a competitive market. The increase in online retail prices is driven by businesses aiming to maximize profits. Specifically, as online channels gain popularity and consumer trust grows, the preference for purchasing drugs through online channels grows. Consumers are willing to pay slightly higher prices for the convenience and rapid access to drugs. Observing this trend, businesses gradually adjust their pricing strategies to capitalize on the opportunity to increase profits by raising online retail prices. This strategy leverages changes in market supply and demand dynamics, optimizing revenue by catering to consumers’ increasing preference for convenience and their acceptance of higher prices. Figure 6 illustrates that as consumer acceptance of online purchases increases, offline channel demand rises while online channel demand gradually decreases. Although this may seem counterintuitive, it can be explained by businesses’ profit-maximization strategies. For online channel demand, the key factor is businesses’ drive to maximize profits. As consumer trust in online channels grows, more consumers prefer purchasing drugs online. To capitalize on this, businesses raise online retail prices. However, when prices exceed consumers’ expectations, overall online channel demand declines. In contrast, for offline channel demand, as acceptance of online channels increases and online retail prices of drugs significantly rise, many consumers opt to purchase drugs offline to avoid higher prices. This highlights the importance of price considerations in consumers’ drug-purchasing decisions. Even if consumers trust and prefer online channels, they shift to more economical offline options when online prices become too high.

Figure 7 indicates that as consumer acceptance of online purchases increases, pharmaceutical manufacturers’ profits rise while offline retailers’ profits gradually decrease. The increase in pharmaceutical manufacturers’ profits can be attributed to several factors. As consumer acceptance of online purchases improves, their preference for online purchases grows. Despite an overall reduction in online demand due to higher online retail prices, the price increases partly offset the decline in demand. This allows pharmaceutical manufacturers to leverage higher consumer acceptance by strategically raising online drug prices, thereby enhancing overall profitability. This demonstrates that pharmaceutical manufacturers can effectively optimize profits by strategically adjusting prices in response to growing trust in online channels. Conversely, offline retailers face a different scenario. As consumer online acceptance increases, more consumers opt to purchase drugs online, leading to a potential loss of market share for offline retailers. To counter this, offline retailers may reduce prices and implement other measures to maintain their market presence. While these strategies might temporarily boost offline channel demand, they typically fail to fully compensate for the losses from price reductions, resulting in an overall decline in offline retailers’ profitability. This highlights the adverse effect of increasing consumer trust in online channels on offline retailers’ profitability. Therefore, offline retailers need to closely monitor consumer preferences and adjust their market strategies accordingly to sustain their profitability.

### 6.3 The impact of different sales models on channel demand and profitability

**Figure 8:**
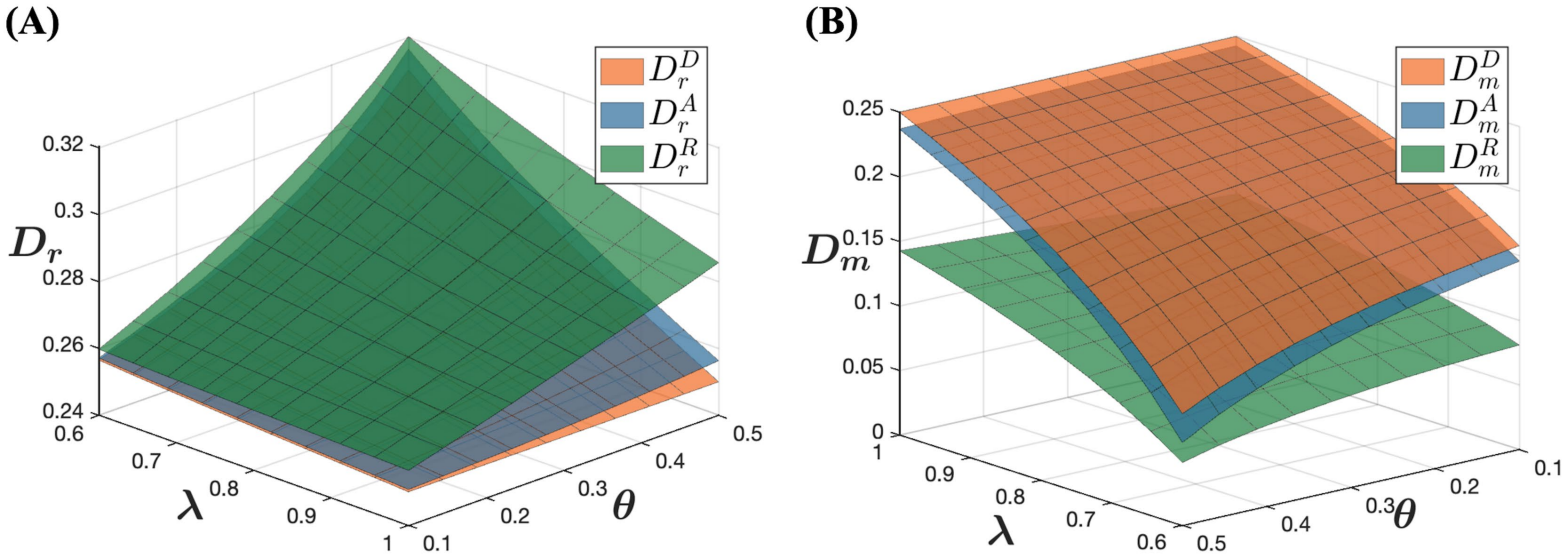
The influence of D, A, and R models on dual-channel demand. **(A)** The influence of D, A, and R models on offline demand. **(B)** The influence of D, A, and R models on online demand.

Figure 8 shows that in the model R, channel demand is highest offline and lowest online, whereas in the model D, channel demand is lowest offline and highest online. This phenomenon can be explained by pricing disparities in these selling models. In all three selling models, offline drug prices are relatively similar, but online retail prices vary significantly. In the model D, drug prices are lowest online, which encourages consumers purchasing behavior, making the online channel more attractive. In contrast, in the model R, higher online prices reduce consumers’ willingness to purchase online, prompting them to prefer buying drugs through physical stores instead. Therefore, in the model R, consumers are less inclined to make online purchases, leading to a greater preference for offline purchasing channels. This distinction in consumer behavior across different sales models highlights the significant impact of pricing strategies on consumer channel preferences and the overall demand distribution between online and offline channels.

**Figure 9:**
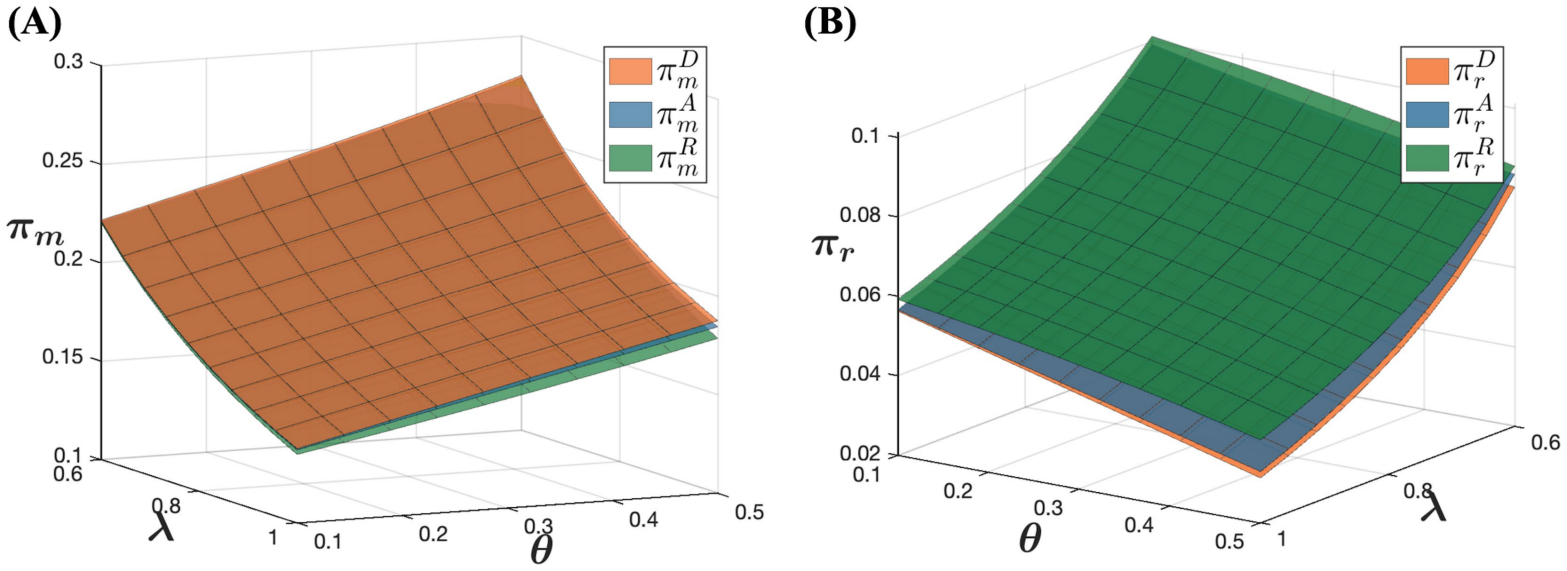
The influence of D, A, and R models on dual-channel profitability. **(A)** The influence of D, A, and R models on pharmaceutical manufacturer’s profit. **(B)** The influence of D, A, and R models on offline retailer’s profit.

According to Figure 9A, in the model D, pharmaceutical manufacturers achieve the highest profit, whereas in the model R, they achieve the lowest profit. This indicates that the choice of sales model significantly impacts pharmaceutical manufacturers’ profit levels. In the model D, pharmaceutical manufacturers engage directly with customers and control the online retail prices of their products. This direct selling approach reduces intermediaries, giving manufacturers greater flexibility in pricing decisions. Additionally, direct selling allows manufacturers to capture sales revenue without incurring additional distribution costs or commission fees, potentially leading to higher profit margins. Conversely, in the model R, pharmaceutical manufacturers wholesale their products to online retailers who set the online retail prices. This indirect sales model limits the manufacturers’ control over pricing and exposes them to competitive pressures from online retailers, squeezing profit margins. Although online retailers attract more traffic and maintain product competitiveness, manufacturers’ overall profit margins are lower. In the model A, manufacturers sell their products online through platforms, paying a commission to the platform. While manufacturers retain some pricing control in this model, resulting in higher profits compared to the model R, their profits are still affected by commission fees. When the percentage of consumers’ out-of-pocket expense is low and consumer trust in online channels is high, manufacturers’ profits in the model R are the lowest. This analysis highlights how different sales models influence pharmaceutical manufacturers’ profitability, emphasizing the trade-offs between direct control over pricing and the benefits of broader market reach through indirect distribution channels.

According to Figure 9B, in the model R, offline retailers achieve the highest profit, whereas in the model D, they achieve the lowest profit. This difference is due to the dynamics within each sales model. In the model R, pharmaceutical manufacturers wholesale drugs to both offline retailers and online retailers. Online retailers typically mark up prices after purchasing drugs at a lower wholesale price to gain additional profit. This price markup reduces the price advantage of online drugs, prompting price-sensitive consumers to prefer offline purchases. Consequently, this enhances the competitiveness and profitability of offline retailers. Conversely, in the model D, pharmaceutical manufacturers sell drugs directly to consumers through online channels and control the online retail prices. As a result, online retail prices are relatively lower, making consumers more inclined to purchase drugs online. This leads to reduced profitability for physical retail stores in the model D. These observations underscore how different sales models impact the profitability of offline retailers, highlighting the trade-offs between pricing control, consumer preferences, and competitive dynamics in the pharmaceutical market.

## 7 Summary and recommendations

### 7.1 Conclusion

This paper examines three distinct selling models for online drug distribution: the dual-channel model for online direct selling, the dual-channel model for online agency selling, and the dual-channel model for online reselling. It investigates optimal pricing decisions under these models, considering medical insurance policy and consumer channel preferences. This paper evaluates how these factors impact pricing, channel demand dynamics, and profitability, providing insights to help pharmaceutical manufacturers select optimal sales channel strategies. Through numerical analysis, this study derives the following conclusions:

1. Medical insurance policy can significantly enhance the profits of pharmaceutical manufacturers and retailers. An increase in the reimbursement ratio boosts overall demand and sales volume for pharmaceutical products, thereby increasing revenues and profits for manufacturers and retailers alike. However, as demand for drugs rises, businesses may adjust pricing strategies to maximize profits, leading to an increase in drug prices. Thus, while medical insurance policy can enhance corporate profits, they also carry the risk of rising drug prices.
2. For pharmaceutical manufacturers, the dual-channel model for direct selling yields maximum profit. In this model, manufacturers bypass intermediaries such as distributors and online retailers, allowing them to sell products directly to consumers. This direct engagement enables manufacturers to maintain higher sales prices and better control over pricing strategies, optimizing profits. For offline retailers, the dual-channel model for online agency selling is most profitable. In this model, manufacturers wholesale drugs to both offline and online retailers. Since online retailers add additional costs to the selling price, cost-sensitive consumers may find that purchasing drugs online is not significantly cheaper than buying them directly from physical stores using medical insurance. Consequently, more consumers opt to purchase drugs from offline retailers.
3. Increasing consumer online acceptance does not necessarily lead to increased demand for pharmaceuticals through these channels; it can yield the opposite effect. While enhanced online acceptance may initially sway more consumers toward online purchases, online retailers often raise drug prices to maximize profits, erasing any pricing advantages. Meanwhile, offline retailers may lower prices to attract consumers and enhance market competitiveness. As a result, despite higher consumer online acceptance in online channels, this does not always translate into increased demand for pharmaceuticals online. Overall, consumer purchasing decisions are influenced by various factors, including pricing, trustworthiness, and shopping experience, in addition to channel preferences.

These findings offer valuable insights for pharmaceutical manufacturers in selecting optimal sales channel strategies, highlighting the trade-offs between direct control over pricing and the benefits of broader market reach through indirect distribution channels. Expanding beyond the context of China, these insights are also applicable to global discussions on dual-channel pharmaceutical supply chains, particularly in countries such as the United States and the United Kingdom. In the U.S., where private healthcare providers play a dominant role, dual-channel models may need to address complexities related to diverse insurance coverages and a highly competitive retail market. In contrast, the U.K.’s National Health Service (NHS) provides a more centralized approach, potentially influencing the acceptance and implementation of dual-channel strategies. By examining these differences, this study offers a foundation for adapting dual-channel approaches under varying regulatory frameworks, providing practical guidance for policymakers and pharmaceutical companies in different healthcare systems.

### 7.2 Strategies

Based on the above conclusions, we offer the following strategies and managerial insights:

1. Develop and implement multi-tiered medical insurance policies

Governments must develop and implement comprehensive, multi-tiered medical insurance policies that balance the interests of pharmaceutical manufacturers, retailers, and consumers. These policies should safeguard the profitability of pharmaceutical manufacturers and retail entities while preventing excessive drug pricing, ensuring that the public’s medication costs remain manageable. The goal is to achieve both economic and social benefits and ensuring the sustainable development of the pharmaceutical industry.

- Improving price transparency: Implement price transparency requirements, obliging pharmaceutical companies to disclose production costs and pricing strategies to allow consumers and retailers to better understand price rationality.
- Optimizing the insurance payment system: Further refine insurance payment standards to ensure that insurance payments are reasonably aligned with drug prices, balancing company profitability and consumer affordability.

2. Enhance consumer acceptance of online pharmaceutical channels

To increase demand for pharmaceuticals through online channels, it is essential to enhance the consumer acceptance of online purchases and establish reasonable pricing strategies. This ensures that consumers perceive genuine price advantages and enjoy a positive shopping experience when purchasing pharmaceuticals online.

- Establish consumer protection mechanisms: Develop industry standards to guarantee the quality and service of online pharmaceutical sales. For example, implement an online pharmaceutical sales compliance certification to ensure that online platforms sell drugs that meet national pharmaceutical standards.
- Personalized recommendations and promotions: Use consumer behavior analytics to design personalized drug recommendations and discount strategies, encouraging consumers to choose online channels.

3. Develop tailored channel expansion strategies for pharmaceutical companies

Large and small-to-medium-sized pharmaceutical companies (SMEs) should adopt customized strategies when expanding into dual-channel markets based on their resources and market position.

- For large pharmaceutical companies: The online direct selling model is recommended. In addition, consider partnerships with medical e-commerce platforms to increase market penetration.
- For small and medium-sized enterprises (SMEs): Before entering online markets, SMEs should ensure sufficient funding, brand recognition, and market reach. Collaborating with established e-commerce platforms can help them gradually enter the online market, reducing upfront investment pressure. SMEs are encouraged to prioritize online consignment models to share channel development costs and lower operational risks.

These strategies aim to optimize the benefits of medical insurance policies, enhance consumer experiences, and tailor channel strategies to the unique needs of different-sized pharmaceutical manufacturers, promoting overall industry sustainability and growth.

### 7.3 Research limitations and future research trends

There are several limitations in this paper. Firstly, the study focuses solely on medical insurance policy and consumer channel preferences, without considering the costs associated with channel establishment and operations. Subsequent studies could delve into these costs and assess their impact on the choice of sales models.

Secondly, this paper confines its examination of medical insurance policy to reimbursement policies, without incorporating factors such as price regulation. Subsequent research could include these elements to provide a more comprehensive analysis. By addressing these limitations, future research can offer a deeper and more nuanced understanding of the dynamics between pharmaceutical manufacturers, retailers, and consumers, thereby enhancing the strategic insights for optimizing sales models and policies in the pharmaceutical industry.

## Data Availability

The original contributions presented in the study are included in the article/Supplementary material, further inquiries can be directed to the corresponding author.
