# Supplementary material for: Research on the selection of OTC drug supply chain sales models: based on medical insurance policy
Source: Front Public Health. 2025 Feb 19;13:1484635. doi: 10.3389/fpubh.2025.1484635 (PMC11879932; doi:10.3389/fpubh.2025.1484635)
Supplement: Supplementary file 1 [file Data_Sheet_1.DOCX]

**Appendix**

**Appendix A**

Using backward induction to solve the model, we first solve the two-stage decision-making problem. We calculate the second partial derivative of the profit function of the offline retailer with respect to $p_{r}^{D}$. The result is $\frac{\partial\pi_{r}^{D}}{\partial p_{r}^{D}}=-\frac{2\lambda}{1-\theta}<0$, indicating that the profit function of the offline retailer is a concave function with respect to $p_{r}^{D}$. This means there exists an optimal offline retail price ${p_{r}^{D}}^{*}$. Setting the first partial derivative of the profit function of the offline retailer with respect to $p_{r}^{D}$ to zero, we obtain ${p_{r}^{D}}^{*}$ as:

$$\begin{aligned} {p_{r}^{D}}^{*}=\frac{1+p_{m}^{D}-\theta+\omega_{r}^{D}\lambda}{2\lambda}\#\left( A.1 \right) \end{aligned}$$

Substituting equation (A.1) into the profit function of the pharmaceutical manufacturer, we derive the Hessian matrix with respect to $\omega_{r}^{D}$ and $p_{m}^{D}$ :

$$\begin{aligned} \left| H \right|=\left[ \begin{matrix} \frac{\lambda}{-1+\theta} & \frac{-1-\lambda}{2(-1+\theta)} \\ \frac{-1-\lambda}{2(-1+\theta)} & -\frac{-2+\theta}{(-1+\theta)\theta} \end{matrix} \right]\#\left( A.2 \right) \end{aligned}$$

From the Hessian matrix, we know that the matrix is negative definite when the second-order leading principal minor is greater than zero, i.e., when $8\lambda-\theta(1+6\lambda+\lambda^{2})>0$ . This condition ensures the existence of an optimal solution for the pharmaceutical manufacturer’s profit function. By substituting equation (A.1) into the pharmaceutical manufacturer’s profit function and setting the first-order derivatives with respect to $\omega_{r}^{D}$ and $p_{m}^{D}$ to zero, we obtain the optimal offline wholesale price ${\omega_{r}^{D}}^{*}$ and the optimal online retail price ${p_{m}^{D}}^{*}$ :

$$\begin{aligned} {\omega_{r}^{D}}^{*}=\frac{\left( -1+\theta\right)\left( 4-\theta+\theta\lambda\right)}{-8\lambda+\theta\left( 1+6\lambda+\lambda^{2} \right)}\# \left( A.3 \right) \end{aligned}$$

$$\begin{aligned} {p_{m}^{D}}^{*}=\frac{\left( -1+\theta\right)\left( \theta+3\theta\lambda\right)}{-8\lambda+\theta\left( 1+6\lambda+\lambda^{2} \right)}\#\left( A.4 \right) \end{aligned}$$

By substituting equations (A.3) and (A.4) into the pharmaceutical manufacturer’s profit function, we obtain the optimal offline retail price ${p_{r}^{D}}^{*}$ :

$$\begin{aligned} {p_{r}^{D}}^{*}=\frac{2\left( 3-4\theta+\theta^{2} \right)}{-8\lambda+\theta\left( 1+6\lambda+\lambda^{2} \right)}\#\left( A.5 \right) \end{aligned}$$

Substituting equations (A.3), (A.4), and (A.5) into the demand function, we get:

$$\begin{aligned} {D_{r}^{D}}^{*}=\frac{\lambda\left( -2+\theta+\theta\lambda\right)}{-8\lambda+\theta\left( 1+6\lambda+\lambda^{2} \right)}\#\left( A.6 \right) \end{aligned}$$

$$\begin{aligned} {D_{m}^{D}}^{*}=\frac{1-3\lambda+2\theta\lambda}{-8\lambda+\theta\left( 1+6\lambda+\lambda^{2} \right)}\#\left( A.7 \right) \end{aligned}$$

Finally, the profit functions of the pharmaceutical manufacturer and the offline retailer are respectively:

$$\begin{aligned} {\pi_{m}^{D}}^{*}=\frac{\left( -1+\theta\right)\left( 1+\theta\lambda\right)}{-8\lambda+\theta\left( 1+6\lambda+\lambda^{2} \right)}\#\left( A.8 \right) \end{aligned}$$

$$\begin{aligned} {\pi_{r}^{D}}^{*}=-\frac{\left( -1+\theta\right)\lambda\left( -2+\theta+\theta\lambda\right)^{2}}{\left( -8\lambda+\theta\left( 1+6\lambda+\lambda^{2} \right) \right)^{2}}\#\left( A.9 \right) \end{aligned}$$

**Appendix B**

The solution process is similar to Appendix A. First, we solve the two-stage decision-making problem by calculating the second partial derivative of the profit function of the offline retailer with respect to $p_{r}^{A}$ . The result is $\frac{\partial\pi_{r}^{A}}{\partial p_{r}^{A}}=-\frac{2\lambda}{1-\theta}<0$ , indicating that the profit function of the offline retailer is concave with respect to $p_{r}^{A}$ . Therefore, there exists an optimal offline retail price ${p_{r}^{A}}^{*}$ . Setting the first partial derivative of the profit function of the offline retailer with respect to $p_{r}^{A}$ to zero, we obtain ${p_{r}^{A}}^{*}$ as:

$$\begin{aligned} {p_{r}^{A}}^{*}=\frac{1+p_{m}^{A}-\theta+\omega_{r}^{A}\lambda}{2\lambda}\#\left( B.1 \right) \end{aligned}$$

Substituting equation (B.1) into the profit function of the pharmaceutical manufacturer, we derive the Hessian matrix with respect to $\omega_{r}^{A}$ and $\omega_{m}^{A}$ :

$$\begin{aligned} \left| H \right|=\left[ \begin{matrix} \frac{\lambda}{-1+\theta} & \frac{-1-\lambda+\gamma\lambda}{2(-1+\theta)} \\ \frac{-1-\lambda+\gamma\lambda}{2(-1+\theta)} & \frac{(-2+\theta)(-1+\gamma)}{(-1+\theta)\theta} \end{matrix} \right]\#\left( B.2 \right) \end{aligned}$$

From the Hessian matrix, we know that it is negative definite when the second-order leading principal minor is greater than zero, i.e., when $-4(-1+\gamma)(-2+\theta)\lambda+\theta{(1+\lambda-\gamma\lambda)}^{2}<0$ . This condition ensures the existence of an optimal solution for the pharmaceutical manufacturer’s profit function. By substituting equation (B.1) into the pharmaceutical manufacturer’s profit function and setting the first-order derivatives with respect to $\omega_{r}^{A}$ and $p_{m}^{A}$ to zero, we obtain the optimal offline wholesale price ${\omega_{r}^{A}}^{*}$ and the optimal online retail price ${p_{m}^{A}}^{*}$ :

$$\begin{aligned} {\omega_{r}^{A}}^{*}=\frac{\left( -1+\gamma\right)\left( -1+\theta\right)\left( -4+\theta+\left( -1+\gamma\right)\theta\lambda\right)}{\left( -1+\gamma\right)\lambda\left( \theta\lambda\left( -1+\gamma\right)-6\theta+8 \right)+\theta}\#\left( B.3 \right) \end{aligned}$$

$$\begin{aligned} {p_{m}^{A}}^{*}=-\frac{\left( -1+\theta\right)\left( -\theta-3\theta\lambda+3\gamma\theta\lambda\right)}{\left( -1+\gamma\right)\lambda\left( \theta\lambda\left( -1+\gamma\right)-6\theta+8 \right)+\theta}\#\left( B.4 \right) \end{aligned}$$

By substituting equations (B.3) and (B.4) into the pharmaceutical manufacturer’s profit function, we obtain the optimal offline retail price ${p_{r}^{A}}^{*}$ :

$$\begin{aligned} {p_{r}^{A}}^{*}=\frac{2\left( -1+\gamma\right)\left( -3+\theta\right)\left( -1+\theta\right)}{\left( -1+\gamma\right)\lambda\left( \theta\lambda\left( -1+\gamma\right)-6\theta+8 \right)+\theta}\#\left( B.5 \right) \end{aligned}$$

Substituting equations (B.3), (B.4), and (B.5) into the demand function, we get:

$$\begin{aligned} {D_{r}^{A}}^{*}=\frac{\left( -1+\gamma\right)\lambda\left( 2+\theta\left( -1+\left( -1+\gamma\right)\lambda\right) \right)}{\left( -1+\gamma\right)\lambda\left( \theta\lambda\left( -1+\gamma\right)-6\theta+8 \right)+\theta}\#\left( B.6 \right) \end{aligned}$$

$$\begin{aligned} {D_{m}^{A}}^{*}=\frac{1-\left( -1+\gamma\right)\left( -3+2\theta\right)\lambda}{\left( -1+\gamma\right)\lambda\left( \theta\lambda\left( -1+\gamma\right)-6\theta+8 \right)+\theta}\#\left( B.7 \right) \end{aligned}$$

Finally, the profit functions of the pharmaceutical manufacturer, the offline retailer, and the online retailer are respectively:

$$\begin{aligned} {\pi_{m}^{A}}^{*}=\frac{\left( -1+\gamma\right)\left( -1+\theta\right)\left( -1+\left( -1+\gamma\right)\theta\lambda\right)}{\left( -1+\gamma\right)\lambda\left( \theta\lambda\left( -1+\gamma\right)-6\theta+8 \right)+\theta}\#\left( B.8 \right) \end{aligned}$$

$$\begin{aligned} {\pi_{r}^{A}}^{*}=-\frac{\left( -1+\gamma\right)^{2}\left( -1+\theta\right)\lambda\left( 2+\theta\left( -1+\left( -1+\gamma\right)\lambda\right) \right)^{2}}{\left( \left( -1+\gamma\right)\lambda\left( \theta\lambda\left( -1+\gamma\right)-6\theta+8 \right)+\theta\right)^{2}}\#\left( B.9 \right) \end{aligned}$$

$$\begin{aligned} {\pi_{t}^{A}}^{*}=\frac{\gamma\left( -1+\theta\right)\theta\left( -1+3\left( -1+\gamma\right)\lambda\right)\left( -1+\left( -1+\gamma\right)\left( -3+2\theta\right)\lambda\right)}{\left( \left( -1+\gamma\right)\lambda\left( \theta\lambda\left( -1+\gamma\right)-6\theta+8 \right)+\theta\right)^{2}}\#\left( B.10 \right) \end{aligned}$$

**Appendix C**

Similarly, we start by solving the two-stage decision-making problem, calculating the second partial derivatives of the offline retailer’s profit function with respect to $p_{r}^{R}$ and $p_{m}^{R}$ . The Hessian matrix is given by:

$$\begin{aligned} \left| H \right|=\left[ \begin{matrix} -\frac{2\lambda}{1-\theta} & \frac{1}{1-\theta} \\ \frac{1}{1-\theta} & -\frac{2}{(-1+\theta)\theta} \end{matrix} \right]\#\left( C.1 \right) \end{aligned}$$

This indicates that when $\left| H \right|>0$ , i.e., when $4\lambda-\theta>0$ , there exist optimal offline retail prices ${p_{r}^{R}}^{*}$ and ${p_{m}^{R}}^{*}$ . Setting the first partial derivatives of the offline retailer’s profit function with respect to $p_{r}^{R}$ and $p_{m}^{R}$ to zero, we obtain:

$$\begin{aligned} {p_{r}^{R}}^{*}=-\frac{2-2\theta+\omega_{m}^{R}+2\lambda\omega_{r}^{R}}{\left( -4+\theta\right)\lambda}\# \left( C.2 \right) \end{aligned}$$

$$\begin{aligned} {p_{m}^{R}}^{*}=-\frac{\theta-\theta^{2}+2\omega_{m}^{R}+\theta\lambda\omega_{r}^{R}}{-4+\theta}\#\left( C.3 \right) \end{aligned}$$

Substituting equations (C.2) and (C.3) into the pharmaceutical manufacturer’s profit function, we derive the Hessian matrix with respect to $\omega_{r}^{R}$ and $\omega_{m}^{R}$ :

$$\begin{aligned} \left| H \right|=\left[ \begin{matrix} \frac{-4\theta\lambda+2\theta^{2}\lambda}{(-4+\theta)(-1+\theta)\theta} & \frac{1+\lambda}{(-4+\theta)(-1+\theta)} \\ \frac{1+\lambda}{(-4+\theta)(-1+\theta)} & \frac{-4+2\theta}{(-4+\theta)(-1+\theta)\theta} \end{matrix} \right]\#\left( C.4 \right) \end{aligned}$$

From the Hessian matrix, we know that it is negative definite when the second-order leading principal minor is greater than zero, i.e., when $16\lambda+4\theta^{2}\lambda-\theta(1+18\lambda+\lambda^{2})>0$ . This condition ensures the existence of an optimal solution for the pharmaceutical manufacturer’s profit function. By substituting equations (C.2) and (C.3) into the pharmaceutical manufacturer’s profit function and setting the first-order derivatives with respect to $\omega_{r}^{R}$ and $\omega_{m}^{R}$ to zero, we obtain the optimal offline wholesale price ${\omega_{r}^{R}}^{*}$ and the optimal online retail price ${\omega_{m}^{R}}^{*}$ :

$$\begin{aligned} {\omega_{r}^{R}}^{*}=-\frac{\left( -1+\theta\right)\left( 8+\theta\left( -3+\lambda\right) \right)}{16\lambda+4\theta^{2}\lambda-\theta\left( 1+18\lambda+\lambda^{2} \right)}\#\left( C.5 \right) \end{aligned}$$

$$\begin{aligned} {\omega_{m}^{R}}^{*}=\frac{2\left( -1+\theta\right)\theta\left( -1+\left( -3+\theta\right)\lambda\right)}{16\lambda+4\theta^{2}\lambda-\theta\left( 1+18\lambda+\lambda^{2} \right)}\#\left( C.6 \right) \end{aligned}$$

By substituting equations (C.5) and (C.6) into the pharmaceutical manufacturer’s profit function, we obtain the optimal offline and online retail prices ${p_{r}^{R}}^{*}$ and ${p_{m}^{R}}^{*}$ :

$$\begin{aligned} {p_{r}^{R}}^{*}=\frac{6\left( 2-3\theta+\theta^{2} \right)}{16\lambda+4\theta^{2}\lambda-\theta\left( 1+18\lambda+\lambda^{2} \right)}\#\left( C.7 \right) \end{aligned}$$

$$\begin{aligned} {p_{m}^{R}}^{*}=\frac{\left( -1+\theta\right)\theta\left( -1+\left( -9+4\theta\right)\lambda\right)}{16\lambda+4\theta^{2}\lambda-\theta\left( 1+18\lambda+\lambda^{2} \right)}\#\left( C.8 \right) \end{aligned}$$

Substituting equations (C.5), (C.6), (C.7), and (C.8) into the demand function, we get:

$$\begin{aligned} {D_{r}^{R}}^{*}=\frac{\lambda\left( -4+\theta\left( 3+\lambda\right) \right)}{-16\lambda-4\theta^{2}\lambda+\theta\left( 1+18\lambda+\lambda^{2} \right)}\#\left( C.9 \right) \end{aligned}$$

$$\begin{aligned} {D_{m}^{R}}^{*}=\frac{1-3\lambda+2\theta\lambda}{\theta-16\lambda+18\theta\lambda-4\theta^{2}\lambda+\theta\lambda^{2}}\#\left( C.10 \right) \end{aligned}$$

Finally, the profit functions of the pharmaceutical manufacturer, the offline retailer, and the online retailer are respectively:

$$\begin{aligned} {\pi_{m}^{R}}^{*}=-\frac{\left( -1+\theta\right)\left( 2+\theta\lambda\right)}{16\lambda+4\theta^{2}\lambda-\theta\left( 1+18\lambda+\lambda^{2} \right)}\#\left( C.11 \right) \end{aligned}$$

$$\begin{aligned} {\pi_{r}^{R}}^{*}=-\frac{\left( -1+\theta\right)\lambda\left( -4+\theta\left( 3+\lambda\right) \right)^{2}}{\left( 16\lambda+4\theta^{2}\lambda-\theta\left( 1+18\lambda+\lambda^{2} \right) \right)^{2}}\#\left( C.12 \right) \end{aligned}$$

$$\begin{aligned} {\pi_{t}^{R}}^{*}=-\frac{\left( -1+\theta\right)\theta\left( 1+\left( -3+2\theta\right)\lambda\right)^{2}}{\left( 16\lambda+4\theta^{2}\lambda-\theta\left( 1+18\lambda+\lambda^{2} \right) \right)^{2}}\#\left( C.13 \right) \end{aligned}$$

**Appendix D**

Based on Appendix A, we have:$p_{r}^{D}-p_{m}^{D}=-\frac{3(-1+\theta)(-2+\theta+\theta\lambda)}{-8\lambda+\theta(1+6\lambda+\lambda^{2})}$. Since $8\lambda-\theta(1+6\lambda+\lambda^{2})>0$, and we can prove that $\left( -1+\theta\right)<0$，$\left( -2+\theta+\theta\lambda\right)<0$, it follows that $p_{r}^{D}-p_{m}^{D}>0$. Similarly, we can infer the following results for other cases.

$p_{r}^{A}-p_{m}^{A}=\frac{\left( -1+\theta\right)\left( -3\left( -2+\theta+\theta\lambda\right)+\gamma\left( -6+\theta\left( 2+3\lambda\right) \right) \right)}{(-1+\gamma)\lambda(\theta\lambda(-1+\gamma)-6\theta+8)+\theta}>0$*.* $p_{r}^{R}-p_{m}^{R}=-\frac{\left( -1+\theta\right)\left( 12+4\theta^{2}\lambda-\theta\left( 7+9\lambda\right) \right)}{16\lambda+4\theta^{2}\lambda-\theta\left( 1+18\lambda+\lambda^{2} \right)}>0$*.*

$D_{r}^{D}-D_{m}^{D}=\frac{\left( -1+\lambda\right)\left( 1+\theta\lambda\right)}{-8\lambda+\theta\left( 1+6\lambda+\lambda^{2} \right)}>0$*.* $D_{r}^{A}-D_{m}^{A}=\frac{\left( 1+\left( -1+\gamma\right)\lambda\right)\left( -1+\left( -1+\gamma\right)\theta\lambda\right)}{(-1+\gamma)\lambda(\theta\lambda(-1+\gamma)-6\theta+8)+\theta}>0$*.*

$D_{r}^{R}-D_{m}^{R}=\frac{\left( 1+\lambda\right)\left( -1+\theta\lambda\right)}{-16\lambda-4\theta^{2}\lambda+\theta\left( 1+18\lambda+\lambda^{2} \right)}>0$*.* $\pi_{m}^{D}-\pi_{r}^{D}=\frac{\left( -1+\theta\right)\left( -4\lambda+\theta\left( 1+2\lambda-11\lambda^{2} \right)+2\theta^{2}\lambda\left( 1+4\lambda+\lambda^{2} \right) \right)}{\left( -8\lambda+\theta\left( 1+6\lambda+\lambda^{2} \right) \right)^{2}}>0$*.*

$\pi_{m}^{A}-\pi_{r}^{A}=\frac{(-1+\gamma)(-1+\theta)(-4(-1+\gamma)\lambda+2(-1+\gamma)\theta^{2}\lambda(1-4(-1+\gamma)\lambda+{(-1+\gamma)}^{2}\lambda^{2})+\theta(-1+2(-1+\gamma)\lambda+11{(-1+\gamma)}^{2}\lambda^{2}))}{{((-1+\gamma)\lambda(\theta\lambda(-1+\gamma)-6\theta+8)+\theta)}^{2}}>0$*.*

$\pi_{m}^{R}-\pi_{r}^{R}=-\frac{2\left( -1+\theta\right)\left( 8\lambda+2\theta^{3}\lambda^{2}-\theta^{2}\lambda\left( 1+12\lambda+\lambda^{2} \right)+\theta\left( -1-6\lambda+11\lambda^{2} \right) \right)}{\left( 16\lambda+4\theta^{2}\lambda-\theta\left( 1+18\lambda+\lambda^{2} \right) \right)^{2}}>0$*.*

**Appendix E**

Calculate the first-order partial derivatives of prices, demand, and profit with respect to the percentage of consumers’ out-of-pocket expense under different sales models:

$\frac{\partial\omega_{r}^{D}}{\partial\lambda}=-\frac{(-1+\theta)(-32+8\theta(4+\lambda)+\theta^{2}(-7-2\lambda+\lambda^{2}))}{{(-8\lambda+\theta(1+6\lambda+\lambda^{2}))}^{2}}<0$.

$\frac{\partial\omega_{r}^{A}}{\partial\lambda}=-\frac{{(-1+\gamma)}^{2}(-1+\theta)(-32+8\theta(4+\lambda-\gamma\lambda)+\theta^{2}(-7+2(-1+\gamma)\lambda+{(-1+\gamma)}^{2}\lambda^{2}))}{{((-1+\gamma)\lambda(\theta\lambda(-1+\gamma)-6\theta+8)+\theta)}^{2}}$<0.

$\frac{\partial\omega_{r}^{R}}{\partial\lambda}=-\frac{\left( -1+\theta\right)\left( -128+12\theta^{3}+16\theta\left( 12+\lambda\right)+\theta^{2}\left( -87-6\lambda+\lambda^{2} \right) \right)}{\left( 16\lambda+4\theta^{2}\lambda-\theta\left( 1+18\lambda+\lambda^{2} \right) \right)^{2}}<0$.

$\frac{\partial p_{r}^{D}}{\partial\lambda}=\frac{4(3-4\theta+\theta^{2})(-4+\theta(3+\lambda))}{{(-8\lambda+\theta(1+6\lambda+\lambda^{2}))}^{2}}$<0. $\frac{\partial p_{r}^{A}}{\partial\lambda}=-\frac{4\left( -1+\gamma\right)^{2}\left( -3+\theta\right)\left( -1+\theta\right)\left( 4+\theta\left( -3+\left( -1+\gamma\right)\lambda\right) \right)}{\left( (-1+\gamma)\lambda(\theta\lambda(-1+\gamma)-6\theta+8)+\theta\right)^{2}}<0$.

$\frac{\partial p_{r}^{R}}{\partial\lambda}=-\frac{12\left( 2-3\theta+\theta^{2} \right)\left( 8+2\theta^{2}-\theta\left( 9+\lambda\right) \right)}{\left( 16\lambda+4\theta^{2}\lambda-\theta\left( 1+18\lambda+\lambda^{2} \right) \right)^{2}}<0$. $\frac{\partial p_{m}^{D}}{\partial\lambda}=-\frac{\left( -1+\theta\right)\theta\left( -8+\theta\left( 3+2\lambda+3\lambda^{2} \right) \right)}{\left( -8\lambda+\theta\left( 1+6\lambda+\lambda^{2} \right) \right)^{2}}<0$.

$\frac{\partial p_{m}^{A}}{\partial\lambda}=-\frac{\left( -1+\theta\right)\theta\left( -1+3\left( -1+\gamma\right)\lambda\right)}{(-1+\gamma)\lambda(\theta\lambda(-1+\gamma)-6\theta+8)+\theta}<0$. $\frac{\partial p_{m}^{R}}{\partial\lambda}=\frac{(-1+\theta)\theta(16+4\theta^{2}\lambda^{2}-\theta(9+2\lambda+9\lambda^{2}))}{{(16\lambda+4\theta^{2}\lambda-\theta(1+18\lambda+\lambda^{2}))}^{2}}<0$.

$\frac{\partial D_{r}^{D}}{\partial\lambda}=\frac{\theta(-2+\theta+2\theta\lambda-6\lambda^{2}+5\theta\lambda^{2})}{{(-8\lambda+\theta(1+6\lambda+\lambda^{2}))}^{2}}<0$.

$\frac{\partial D_{r}^{A}}{\partial\lambda}=-\frac{\left( -1+\gamma\right)\theta\left( \theta-2\left( -1+\gamma\right)\theta\lambda+5\left( -1+\gamma\right)^{2}\theta\lambda^{2}-2\left( 1+3\left( -1+\gamma\right)^{2}\lambda^{2} \right) \right)}{\left( (-1+\gamma)\lambda(\theta\lambda(-1+\gamma)-6\theta+8)+\theta\right)^{2}}<0$.

$\frac{\partial D_{r}^{R}}{\partial\lambda}=\frac{\theta(-4\theta^{2}\lambda^{2}-4(1+3\lambda^{2})+\theta(3+2\lambda+15\lambda^{2}))}{{(16\lambda+4\theta^{2}\lambda-\theta(1+18\lambda+\lambda^{2}))}^{2}}<0$.

$\frac{\partial D_{m}^{D}}{\partial\lambda}=\frac{8-2\theta^{2}(-1+\lambda^{2})+\theta(-9-2\lambda+3\lambda^{2})}{{(-8\lambda+\theta(1+6\lambda+\lambda^{2}))}^{2}}>0$.

$\frac{\partial D_{m}^{A}}{\partial\lambda}=\frac{(-1+\gamma)(-8+\theta(9-2(-1+\gamma)\lambda-3{(-1+\gamma)}^{2}\lambda^{2})+2\theta^{2}(-1+{(-1+\gamma)}^{2}\lambda^{2}))}{{((-1+\gamma)\lambda(\theta\lambda(-1+\gamma)-6\theta+8)+\theta)}^{2}}>0$.

$\frac{\partial D_{m}^{R}}{\partial\lambda}=\frac{16-2\theta^{2}(-3+\lambda^{2})+\theta(-21-2\lambda+3\lambda^{2})}{{(16\lambda+4\theta^{2}\lambda-\theta(1+18\lambda+\lambda^{2}))}^{2}}>0$.

$\frac{\partial\pi_{m}^{D}}{\partial\lambda}=-\frac{\left( -1+\theta\right)\left( -8+2\theta\left( 3+\lambda\right)+\theta^{2}\left( -1+\lambda^{2} \right) \right)}{\left( -8\lambda+\theta\left( 1+6\lambda+\lambda^{2} \right) \right)^{2}}<0$.

$\frac{\partial\pi_{m}^{A}}{\partial\lambda}=-\frac{{(-1+\gamma)}^{2}(-1+\theta)(-8+\theta(6-2(-1+\gamma)\lambda)+\theta^{2}(-1+{(-1+\gamma)}^{2}\lambda^{2}))}{{((-1+\gamma)\lambda(\theta\lambda(-1+\gamma)-6\theta+8)+\theta)}^{2}}$<0.

$\frac{\partial\pi_{m}^{R}}{\partial\lambda}=-\frac{\left( -1+\theta\right)\left( -32+4\theta\left( 9+\lambda\right)+\theta^{2}\left( -9+\lambda^{2} \right) \right)}{\left( 16\lambda+4\theta^{2}\lambda-\theta\left( 1+18\lambda+\lambda^{2} \right) \right)^{2}}<0$.

$\frac{\partial\pi_{r}^{D}}{\partial\lambda}=\frac{(-1+\theta)(-32\lambda+\theta^{3}{(-1+\lambda)}^{3}(1+\lambda)-4\theta^{2}(-1+6\lambda+3\lambda^{2})+4\theta(-1+14\lambda+3\lambda^{2}))}{{(-8\lambda+\theta(1+6\lambda+\lambda^{2}))}^{3}}<0$.

$\frac{\partial\pi_{r}^{A}}{\partial\lambda}=\frac{{(-1+\gamma)}^{2}(-1+\theta)(2+\theta(-1+(-1+\gamma)\lambda))(16(-1+\gamma)\lambda+\theta^{2}{(1+(-1+\gamma)\lambda)}^{3}-2\theta(1+10(-1+\gamma)\lambda+{(-1+\gamma)}^{2}\lambda^{2}))}{{((-1+\gamma)\lambda(\theta\lambda(-1+\gamma)-6\theta+8)+\theta)}^{3}}$<0.

$\frac{\partial\pi_{r}^{R}}{\partial\lambda}=$

$-\frac{\left( -1+\theta\right)\left( -256\lambda+4\theta^{4}\lambda\left( -9+\lambda^{2} \right)-24\theta^{2}\left( -1+26\lambda+3\lambda^{2} \right)+16\theta\left( -1+42\lambda+3\lambda^{2} \right)+\theta^{3}\left( -9+246\lambda+24\lambda^{2}-6\lambda^{3}+\lambda^{4} \right) \right)}{\left( 16\lambda+4\theta^{2}\lambda-\theta\left( 1+18\lambda+\lambda^{2} \right) \right)^{3}}<0$.

**Appendix F**

Calculate the first-order partial derivatives of prices, demand, and profit with respect to the consumer acceptance of online purchases under different sales models:

$\frac{\partial\omega_{r}^{D}}{\partial\theta}=\frac{(-1+\lambda)(-4+12\lambda-16\theta\lambda+\theta^{2}(1+6\lambda+\lambda^{2}))}{{(-8\lambda+\theta(1+6\lambda+\lambda^{2}))}^{2}}$. When $-4+12\lambda-16\theta\lambda+\theta^{2}(1+6\lambda+\lambda^{2}>0$, $\frac{\partial\omega_{r}^{D}}{\partial\theta}<0$. Solving $-4+12\lambda-16\theta\lambda+\theta^{2}(1+6\lambda+\lambda^{2}=0$, gives $\theta<\frac{2(4\lambda+\sqrt{1+3\lambda-\lambda^{2}-3\lambda^{3}})}{1+6\lambda+\lambda^{2}}$, $\frac{\partial\omega_{r}^{D}}{\partial\theta}<0$.

$\frac{\partial\omega_{r}^{A}}{\partial\lambda}=\frac{(-1+\gamma)(1+(-1+\gamma)\lambda)(16(-1+\gamma)\theta\lambda-4(1+3(-1+\gamma)\lambda)+\theta^{2}(1-6(-1+\gamma)\lambda+{(-1+\gamma)}^{2}\lambda^{2}))}{{((-1+\gamma)\lambda(\theta\lambda(-1+\gamma)-6\theta+8)+\theta)}^{2}}$*.*

When $16\left( -1+\gamma\right)\theta\lambda-4\left( 1+3\left( -1+\gamma\right)\lambda\right)+\theta^{2}\left( 1-6\left( -1+\gamma\right)\lambda+\left( -1+\gamma\right)^{2}\lambda^{2} \right)>0$,

$\frac{\partial\omega_{r}^{A}}{\partial\lambda}<0$.Solving$16\left( -1+\gamma\right)\theta\lambda-4\left( 1+3\left( -1+\gamma\right)\lambda\right)+\theta^{2}\left( 1-6\left( -1+\gamma\right)\lambda+\left( -1+\gamma\right)^{2}\lambda^{2} \right)=0$, gives$\theta<\frac{16\lambda-16\gamma\lambda+\sqrt{{(-16\lambda+16\gamma\lambda)}^{2}-4(-4+12\lambda-12\gamma\lambda)(1+6\lambda-6\gamma\lambda+\lambda^{2}-2\gamma\lambda^{2}+\gamma^{2}\lambda^{2})}}{2(1+6\lambda-6\gamma\lambda+\lambda^{2}-2\gamma\lambda^{2}+\gamma^{2}\lambda^{2})}$时$\frac{\partial\omega_{r}^{A}}{\partial\lambda}<0$.

$\frac{\partial\omega_{r}^{R}}{\partial\theta}=\frac{\left( -1+\lambda\right)\left( -8+24\lambda-32\theta\lambda+\theta^{2}\left( 3+12\lambda+\lambda^{2} \right) \right)}{\left( 16\lambda+4\theta^{2}\lambda-\theta\left( 1+18\lambda+\lambda^{2} \right) \right)^{2}}$. When$-8+24\lambda-32\theta\lambda+\theta^{2}\left( 3+12\lambda+\lambda^{2} \right)>0$, $\frac{\partial\omega_{r}^{R}}{\partial\theta}<0$.Solving$-8+24\lambda-32\theta\lambda+\theta^{2}\left( 3+12\lambda+\lambda^{2} \right)=0$, gives$\theta<\frac{2\left( 8\lambda+\sqrt{6}\sqrt{1+\lambda-\lambda^{2}-\lambda^{3}} \right)}{3+12\lambda+\lambda^{2}}$, $\frac{\partial\omega_{r}^{R}}{\partial\theta}<0.$

$\frac{\partial p_{r}^{D}}{\partial\theta}=\frac{6-28\lambda+32\theta\lambda+6\lambda^{2}-2\theta^{2}(1+6\lambda+\lambda^{2})}{{(-8\lambda+\theta(1+6\lambda+\lambda^{2}))}^{2}}$<0.

$\frac{\partial p_{r}^{A}}{\partial\theta}=\frac{2(-1+\gamma)(-3-14(-1+\gamma)\lambda+16(-1+\gamma)\theta\lambda-3{(-1+\gamma)}^{2}\lambda^{2}+\theta^{2}(1-6(-1+\gamma)\lambda+{(-1+\gamma)}^{2}\lambda^{2}))}{{((-1+\gamma)\lambda(\theta\lambda(-1+\gamma)-6\theta+8)+\theta)}^{2}}<0$.

$\frac{\partial p_{r}^{R}}{\partial\theta}=\frac{96\theta\lambda+12(1-6\lambda+\lambda^{2})-6\theta^{2}(1+6\lambda+\lambda^{2})}{{(16\lambda+4\theta^{2}\lambda-\theta(1+18\lambda+\lambda^{2}))}^{2}}$<0. $\frac{\partial p_{m}^{D}}{\partial\theta}=\frac{(1+3\lambda)(8\lambda-16\theta\lambda+\theta^{2}(1+6\lambda+\lambda^{2}))}{{(-8\lambda+\theta(1+6\lambda+\lambda^{2}))}^{2}}>0$.

$\frac{\partial p_{m}^{A}}{\partial\theta}=-\frac{\left( -1+3\left( -1+\gamma\right)\lambda\right)\left( -8\left( -1+\gamma\right)\lambda+16\left( -1+\gamma\right)\theta\lambda+\theta^{2}\left( 1-6\left( -1+\gamma\right)\lambda+\left( -1+\gamma\right)^{2}\lambda^{2} \right) \right)}{\left( (-1+\gamma)\lambda(\theta\lambda(-1+\gamma)-6\theta+8)+\theta\right)^{2}}>0$.

$\frac{\partial p_{m}^{R}}{\partial\theta}=\frac{16\theta^{4}\lambda^{2}+16\lambda(1+9\lambda)-32\theta\lambda(1+13\lambda)-8\theta^{3}\lambda(1+18\lambda+\lambda^{2})+\theta^{2}(1+27\lambda+391\lambda^{2}+13\lambda^{3})}{{(16\lambda+4\theta^{2}\lambda-\theta(1+18\lambda+\lambda^{2}))}^{2}}>0$.

$\frac{\partial D_{r}^{D}}{\partial\theta}=\frac{2\lambda(1+2\lambda-3\lambda^{2})}{{(-8\lambda+\theta(1+6\lambda+\lambda^{2}))}^{2}}>0$. $\frac{\partial D_{r}^{A}}{\partial\theta}=\frac{2(-1+\gamma)\lambda(-1+2(-1+\gamma)\lambda+3{(-1+\gamma)}^{2}\lambda^{2})}{{((-1+\gamma)\lambda(\theta\lambda(-1+\gamma)-6\theta+8)+\theta)}^{2}}>0$.

$\frac{\partial D_{r}^{R}}{\partial\theta}=\frac{4\lambda(1+(6-8\theta+3\theta^{2})\lambda+(-3+\theta^{2})\lambda^{2})}{{(16\lambda+4\theta^{2}\lambda-\theta(1+18\lambda+\lambda^{2}))}^{2}}>0$. $\frac{\partial D_{m}^{D}}{\partial\theta}=\frac{-1-3\lambda+\lambda^{2}+3\lambda^{3}}{{(-8\lambda+\theta(1+6\lambda+\lambda^{2}))}^{2}}<0$.

$\frac{\partial D_{m}^{A}}{\partial\theta}=\frac{-1+3(-1+\gamma)\lambda+{(-1+\gamma)}^{2}\lambda^{2}-3{(-1+\gamma)}^{3}\lambda^{3}}{{((-1+\gamma)\lambda(\theta\lambda(-1+\gamma)-6\theta+8)+\theta)}^{2}}<0$. $\frac{\partial D_{m}^{R}}{\partial\theta}=\frac{-1+(-15+8\theta)\lambda+(21-24\theta+8\theta^{2})\lambda^{2}+3\lambda^{3}}{{(16\lambda+4\theta^{2}\lambda-\theta(1+18\lambda+\lambda^{2}))}^{2}}<0$.

$\frac{\partial\pi_{m}^{D}}{\partial\theta}=\frac{1+(-2+\theta^{2})\lambda+(9-16\theta+6\theta^{2})\lambda^{2}+\theta^{2}\lambda^{3}}{{(-8\lambda+\theta(1+6\lambda+\lambda^{2}))}^{2}}>0$.

$\frac{\partial\pi_{m}^{A}}{\partial\theta}=\frac{(-1+\gamma)(-1+(-1+\gamma)(-2+\theta^{2})\lambda-{(-1+\gamma)}^{2}(9-16\theta+6\theta^{2})\lambda^{2}+{(-1+\gamma)}^{3}\theta^{2}\lambda^{3})}{{((-1+\gamma)\lambda(\theta\lambda(-1+\gamma)-6\theta+8)+\theta)}^{2}}>0$.

$\frac{\partial\pi_{m}^{R}}{\partial\theta}=\frac{2+(4-16\theta+9\theta^{2})\lambda+2(9-16\theta+7\theta^{2})\lambda^{2}+\theta^{2}\lambda^{3}}{{(16\lambda+4\theta^{2}\lambda-\theta(1+18\lambda+\lambda^{2}))}^{2}}>0$.

$\frac{\partial\pi_{r}^{D}}{\partial\theta}=-\frac{\lambda\left( -24\theta^{2}\lambda\left( 1+\lambda\right)^{2}+\theta^{3}\left( 1+\lambda\right)^{2}\left( 1+6\lambda+\lambda^{2} \right)-8\left( -1+2\lambda+3\lambda^{2} \right)+4\theta\left( -2+7\lambda+16\lambda^{2}+3\lambda^{3} \right) \right)}{\left( -8\lambda+\theta\left( 1+6\lambda+\lambda^{2} \right) \right)^{3}}<0$.

$\frac{\partial\pi_{r}^{A}}{\partial\theta}=\frac{(\gamma(-1+3(-1+\gamma)\lambda)(48{(-1+\gamma)}^{2}\theta^{2}\lambda^{2}+8(-1+\gamma)\lambda(1+3(-1+\gamma)\lambda)+2(-1+\gamma)\theta^{3}\lambda(1-6(-1+\gamma)\lambda+{(-1+\gamma)}^{2}\lambda^{2})-\theta(1+13(-1+\gamma)\lambda+63{(-1+\gamma)}^{2}\lambda^{2}+3{(-1+\gamma)}^{3}\lambda^{3})))}{{((-1+\gamma)\lambda(\theta\lambda(-1+\gamma)-6\theta+8)+\theta)}^{3}}$<0.

$\frac{\partial\pi_{r}^{R}}{\partial\theta}=\frac{(\lambda(4\theta^{4}\lambda{(3+\lambda)}^{2}-48\theta^{2}\lambda(-1+4\lambda+\lambda^{2})-32(-1+2\lambda+3\lambda^{2})+8\theta(-5+9\lambda+33\lambda^{2}+3\lambda^{3})+\theta^{3}(9-96\lambda+6\lambda^{2}+16\lambda^{3}+\lambda^{4})))}{{(16\lambda+4\theta^{2}\lambda-\theta(1+18\lambda+\lambda^{2}))}^{3}}<0$*.*
